# Association between trace metal element concentrations in human blood plasma and early MAR embryological outcomes: a couple-based prospective cohort study

**DOI:** 10.1093/hropen/hoaf034

**Published:** 2025-06-10

**Authors:** Yawen Cao, Shuangshuang Bao, Qianhui Yang, Yaning Sun, Yanlan Tang, Wei Ju, Junjun Liu, Wenbin Fang, Xuemei Wang, Caiyun Wu, Chaojie Li, Peng Zhu, Shanshan Shao, Fangbiao Tao, Guixia Pan

**Affiliations:** Department of Epidemiology and Biostatistics, School of Public Health, Anhui Medical University, Hefei, China; MOE Key Laboratory of Population Health Across Life Cycle (Anhui Medical University), Ministry of Education of the People’s Republic of China, Hefei, China; Anhui Provincial Key Laboratory of Environment and Population Health Across the Life Course (Anhui Medical University), Anhui Medical University, Hefei, China; MOE Key Laboratory of Population Health Across Life Cycle (Anhui Medical University), Ministry of Education of the People’s Republic of China, Hefei, China; Anhui Provincial Key Laboratory of Environment and Population Health Across the Life Course (Anhui Medical University), Anhui Medical University, Hefei, China; Department of Maternal, Child and Adolescent Health, School of Public Health, Anhui Medical University, Hefei, China; MOE Key Laboratory of Population Health Across Life Cycle (Anhui Medical University), Ministry of Education of the People’s Republic of China, Hefei, China; Anhui Provincial Key Laboratory of Environment and Population Health Across the Life Course (Anhui Medical University), Anhui Medical University, Hefei, China; Department of Maternal, Child and Adolescent Health, School of Public Health, Anhui Medical University, Hefei, China; Department of Epidemiology and Biostatistics, School of Public Health, Anhui Medical University, Hefei, China; Department of Maternal, Child and Adolescent Health, School of Public Health, Anhui Medical University, Hefei, China; Department of Occupational Health and Environmental Health, School of Public Health, Anhui Medical University, Hefei, China; Department of Maternal, Child and Adolescent Health, School of Public Health, Anhui Medical University, Hefei, China; Department of Epidemiology and Biostatistics, School of Public Health, Anhui Medical University, Hefei, China; Reproductive Medicine Center, The 901th Hospital of the Joint Logistics Support Force of People’s Liberation Army, Hefei, Anhui, China; Reproductive Medicine Center, The 901th Hospital of the Joint Logistics Support Force of People’s Liberation Army, Hefei, Anhui, China; NHC Key Laboratory of Birth Defects Prevention, Zhengzhou, Henan, China; Department of Maternal, Child and Adolescent Health, School of Public Health, Anhui Medical University, Hefei, China; MOE Key Laboratory of Population Health Across Life Cycle (Anhui Medical University), Ministry of Education of the People’s Republic of China, Hefei, China; Anhui Provincial Key Laboratory of Environment and Population Health Across the Life Course (Anhui Medical University), Anhui Medical University, Hefei, China; Department of Maternal, Child and Adolescent Health, School of Public Health, Anhui Medical University, Hefei, China; NHC Key Laboratory of Birth Defects Prevention, Zhengzhou, Henan, China; MOE Key Laboratory of Population Health Across Life Cycle (Anhui Medical University), Ministry of Education of the People’s Republic of China, Hefei, China; Anhui Provincial Key Laboratory of Environment and Population Health Across the Life Course (Anhui Medical University), Anhui Medical University, Hefei, China; Department of Maternal, Child and Adolescent Health, School of Public Health, Anhui Medical University, Hefei, China; Department of Epidemiology and Biostatistics, School of Public Health, Anhui Medical University, Hefei, China; MOE Key Laboratory of Population Health Across Life Cycle (Anhui Medical University), Ministry of Education of the People’s Republic of China, Hefei, China; Anhui Provincial Key Laboratory of Environment and Population Health Across the Life Course (Anhui Medical University), Anhui Medical University, Hefei, China

**Keywords:** trace metal element, couple, infertility, assisted reproductive technology, *in vitro* fertilization

## Abstract

**STUDY QUESTION:**

What are the effects of plasma trace metal element exposure on early embryological outcomes of IVF in couples?

**SUMMARY ANSWER:**

Exposure to plasma trace metal elements before treatment is associated with early embryological outcomes of IVF in couples and both partners, with both harmful and beneficial effects on embryonic development.

**WHAT IS KNOWN ALREADY:**

Trace metal element exposure is one of the strongest determinants of IVF outcomes, but existing studies have certain limitations, such as the limited range of trace metal elements considered, and most have focused only on maternal exposure, overlooking the contribution of paternal exposure. Few studies have explored the association between trace metal elements and early embryological outcomes of IVF from the couples’ perspective.

**STUDY DESIGN, SIZE, DURATION:**

This couple-based prospective cohort study included a total of 1071 couples who underwent 1369 IVF treatment cycles between December 2020 and August 2023.

**PARTICIPANTS/MATERIALS, SETTING, METHODS:**

Plasma concentrations of 21 trace metal elements were measured by an inductively coupled plasma mass spectrometer. Early IVF embryological outcomes included two-pronuclear (2PN) zygote numbers, best-quality embryo numbers, fertilization rates, and blastocyst numbers. Elastic net regression was employed to identify trace metal elements associated with early IVF embryological outcomes in both partners and couples. K-medoids clustering was used to identify the exposure patterns of trace metal elements in couples and both partners. Joint effects of trace metal mixtures were evaluated using quantile-based g-computation (QGC) and group-weighted quantile sum (groupWQS), while independent effects of individual trace metal element were assessed using the generalized linear mixed model.

**MAIN RESULTS AND THE ROLE OF CHANCE:**

In our study, the mean (SD) age was 32.60 (5.22) years for females and 33.79 (5.89) for males. The detection rates for all elements, except for beryllium (Be), exceeded 90%. High exposure to trace metal element mixtures in couples and male partners was associated with decreased numbers of best-quality embryos and blastocysts. Using QGC and groupWQS, we identified both harmful and beneficial metal mixtures that influence successful embryo development. Additionally, specific plasma trace metals such as iron (Fe), lithium (Li), strontium (Sr), and molybdenum (Mo) were positively associated with embryological outcomes, while metals like silver (Ag) and thallium (Tl) had adverse effects.

**LIMITATIONS, REASONS FOR CAUTION:**

We were limited by assessing plasma trace metal elements at a single time point, focusing only on fresh embryo transfer cycles, and being unable to control for unmeasured confounding factors (e.g. psychological factors and self-reported health conditions). Moreover, since our study population was couples undergoing IVF, the extrapolation of our results require caution.

**WIDER IMPLICATIONS OF THE FINDINGS:**

These findings highlight the importance of implementing preconception trace element screening and targeted trace element interventions for couples planning to conceive, as a strategy to optimize reproductive health and IVF outcomes.

**STUDY FUNDING/COMPETING INTEREST(S):**

This work was supported by the National Key Research and Development Program of China (No. 2018YFC1004201), the National Natural Science Foundation of China (No. 82304159), and Open Research Fund of National Health Commission Key Laboratory of Birth Defects Prevention & Henan Key Laboratory of Population Defects Prevention (No. ZD202310). All authors declare no conflict of interest.

**TRIAL REGISTRATION NUMBER:**

N/A.

WHAT DOES THIS MEAN FOR PATIENTS? Humans may take in trace metal elements through diet, water, or air, leading to their accumulation in the human body. Previous studies have indicated that the levels of trace metal elements in both males and females can influence the success of IVF. However, there has been limited research exploring the effects of trace metal concentrations on early embryo development in IVF from the couple’s perspective. Consequently, this study utilized a couple-based prospective cohort of 1071 couples undergoing 1369 IVF cycles to assess the impact of trace metals on early embryo development in IVF. The results suggested that some trace metals, such as iron (Fe), lithium (Li), strontium (Sr), and molybdenum (Mo) may enhance the chances of successful fertilization and embryo development, while other metals, such as silver (Ag) and thallium (Tl) may negatively affect these outcomes. Given the importance of these trace metals in early embryo development, our study suggests that it could be valuable for couples to consider screening for these metals or even explore the option of supportive therapies that regulate metal levels before starting family planning or IVF treatments. Taking some steps, such as optimizing the balance of trace metals in the body, may help enhance fertility and improve IVF success rates.

## Introduction

Infertility is a reproductive system disease caused by various factors, including male factors, female factors, and other unexplained reasons. According to the latest data from the World Health Organization (WHO), approximately one in six individuals of reproductive age globally suffer infertility at some time in their life. However, in contrast with developed countries, the infertility rate in China has been reported to be as high as 17.6%, affecting approximately 33 million couples of reproductive age (Feng and Chen, 2024). IVF, as a medically assisted reproduction technology, is an effective approach to infertility treatment. With the progress and development of IVF, the clinical pregnancy rate has gradually improved in recent years, although the live birth rate is still low, at only about 35% (Sunderam *et al.*, 2022; Deng *et al.*, 2023). To the best of our knowledge, most research focuses on the clinical outcomes of IVF (Chu *et al.*, 2022; Jin *et al.*, 2022; Li *et al.*, 2022). However, early embryological outcomes of IVF, such as the number of two-pronuclear (2PN) zygotes, fertilization rate, and blastocyst numbers, are essential in IVF treatment (Polyzos *et al.*, 2018; Pons *et al.*, 2023). Identifying factors which influence early embryo development could enhance IVF clinical outcomes (Ajduk and Zernicka-Goetz, 2013).

Several studies have suggested that environmental factors are significant in affecting reproductive health and embryo development (Penzias, 2012; Wu *et al.*, 2020; Jin *et al.*, 2022). Notably, trace metal elements play an indispensable role in both embryo development and reproductive health (Kim *et al.*, 2014; Wu *et al.*, 2020; Li *et al.*, 2022). The general population may take in these trace metal elements through diet, water, or air, leading to their accumulation in the human body. The concentration of trace elements in the human body could exert beneficial or harmful effects on the reproductive system. In men, there have been some studies demonstrating that certain metals such as lead (Pb), zinc (Zn), and cadmium (Cd) may affect sperm production, concentration, and motility (Wang *et al.*, 2017; Allouche-Fitoussi and Breitbart, 2020). Lead and cadmium also have been shown to potentially disrupt women’s menstrual cycles (Dutta *et al.*, 2022), impair ovarian function (Tian *et al.*, 2024), and alter female hormone levels (Wang *et al.*, 2023), subsequently affecting the reproductive health of women. It should be noted that the above studies only considered several common metallic elements, which may not comprehensively reflect the association between metallic elements and reproductive health. Generally, embryo formation and development are impacted by the concentrations of trace metal elements in both partners.

However, the majority of studies on the relationship between trace metal elements and reproductive health only consider one partner of the couple (either male or female), rather than both (Wu *et al.*, 2020; Wang *et al.*, 2021; Li *et al.*, 2022; Shi *et al.*, 2023). Additionally, most of these studies have been limited to maternal exposure only and have overlooked the contribution of paternal exposure. It is well known that normal fertilization of the oocyte and normal development of the embryo are determined by the couple. Importantly, while it has been suggested that trace metal element concentrations in both partners might affect embryo development, few studies have emphasized the relationship between trace metal elements and embryo development from the perspectives of couples.

To bridge the above knowledge gaps, we explored the relationship between the concentrations of 21 trace metal elements in plasma and early embryological outcomes of IVF using a couple-based approach in a multi-center prospective cohort.

## Materials and methods

### Population and study design

From December 2020 to August 2023, we conducted a prospective cohort study on the Reproductive Health of Childbearing Couples-Anhui Cohort (RHCC-AC) Infertility Cohort. The cohort enrolled couples who underwent their ART treatment at the Reproductive Center of Maternal and Child Health Care Hospital in Ma’anshan and the 901th Hospital of the Joint Logistics Support Force of the People’s Liberation Army. Inclusion criteria included: (i) women aged 20–49 years old and men aged 22–49 years old; (ii) couples diagnosed with infertility (failure to achieve clinical pregnancy after at least 1 year of unprotected intercourse); (iii) couples with IVF indications including female factors (such as tubal factors or ovulation failure), male factors, or unexplained infertility. Detailed questionnaires were used at enrollment to collect information on demographic, lifestyle, reproduction, and medical history from participants. Clinical data regarding IVF outcomes were obtained from the electronic medical records system. This study was approved by the Ethics Committee of Anhui Medical University (No. 20189999), and all participants provided written informed consent.

The flowchart of study participants is shown in Supplementary Fig. S1. A total of 2437 couples were enrolled in our cohort. Specifically, couples were excluded if they were missing electronic medical records (n = 796) or had not undergone ART treatment (n = 253). Also, couples with abnormal karyotypes (n = 72) and abnormal fertilization, no oocytes retrieved or no embryos available for transfer (n = 41) were excluded. We further excluded couples who did not provide plasma samples for exposure measurement (n = 204). Finally, there were 1071 couples with 1369 cycles available for the current analysis.

### Sample collection

After completing the baseline questionnaire, laboratory physicians used anticoagulant blood collection tubes to draw blood from the cubital veins of both partners. The blood was then transferred into tubes containing anticoagulants. Subsequently, the blood samples were centrifuged at 2500×g for 10 min. After centrifugation, the supernatant was collected and divided into 2 ml Eppendorf tubes. The samples were stored at −80°C until analysis.

### Trace metal element measurements

We employed a direct dilution method, diluting 200 µl of plasma samples 20-fold with 0.05% nitric acid, 0.05% Triton X-100, and 10 µg/l Au, to measure concentrations of 21 trace metal elements in plasma using an inductively coupled plasma mass spectrometer iCAP-TQ (Thermo Fisher Scientific, Waltham, MA, USA) (ICP-MS). Each sample underwent triplicate measurements to ensure robust results. Detection was performed using AccuStandard multi-element calibration solution (ICP-MS-CAL2-1, 10 µg/ml) and multi-element internal standard solution (ICP-MS-200.8-IS-1, 10 µg/ml), selecting internal standards based on mass similarity principles. The linear range for most trace metals was 0.01∼20 ng/l, except for iron (Fe), copper (Cu), Zn, and magnesium (Mg). Calibration curves for all 21 elements exhibited linear correlation coefficients >0.999. Recovery rates for the 21 plasma metals ranged from 92.96% to 108.38%, with intra-day and inter-day precision ranging from 1.67% to 11.67%. Limits of detection (LOD) for the 21 plasma trace metals and their respective internal standards are provided in Supplementary Table S1; concentrations below LOD were assigned LOD/√2 values for analysis. In subsequent statistical analyses, only trace metal elements detected in >50% of individual partners were included.

### IVF procedure and outcome assessment

In clinical practice, IVF involves four main steps. First, an appropriate ovarian stimulation protocol is chosen based on the patient’s ovarian function and menstrual cycle, which includes long protocols, antagonist protocols, or others. Once the patient develops two or more mature follicles, each exceeding 18 mm in diameter, they receive an injection of hCG. Oocyte retrieval surgery is performed 34–36 h after the hCG injection. Concurrently, the male partner provides fresh semen collected via masturbation or percutaneous epididymal sperm aspiration. Following clinical evaluation, either IVF or ICSI is conducted. ICSI is a specific form of IVF in which a single sperm is injected directly into an oocyte for fertilization. After successful fertilization, high-quality embryos are chosen for immediate transfer, while the remaining embryos are frozen. These frozen embryos are later thawed and transferred into the patient’s uterus during a subsequent cycle with a prepared endometrium.

Our study focused on early embryological outcomes of IVF, including numbers of zygotes with 2PN, fertilization rates, numbers of best-quality embryos, and blastocyst numbers. The fertilization rates are expressed as the ratio of 2PN numbers to the total number of fertilized oocytes. Embryos with four cells on Day 2 or 7∼9 cells on Day 3 post-retrieval, no multinucleation, and less than 20% fragmentation are defined as best-quality embryos. Blastocyst numbers refer to the number of embryos developing to a blastocyst with fluid and cavity compartments.

### Statistical analyses

The demographic and clinical characteristics of the study population were described as median (IQR: interquartile range) or mean (SD) for continuous variables and frequency (proportion) for categorical variables. We utilized Student’s *t*-test for normally distributed variables and the chi-square test for categorical variables. In the following analysis, we performed natural logarithm transformation on the concentrations of 21 trace metal elements in plasma to improve normality. Spearman correlation analysis was conducted to explore correlations among trace metal elements within couples.

Elastic net regression (ENR) was used to identify trace metal elements associated with early embryological outcomes of IVF in both male and female partners. Using the mean trace metal element concentrations per couple as a representation of couple-based exposure (Liu *et al.*, 2022), we determined the joint effects and individual effects between these trace metal elements and early embryological outcomes of IVF. To better capture the impact of trace metal element exposure patterns on the outcome, we used K-medoids clustering to classify couples and both partners into different exposure groups. Each partner was classified into the low-exposure or high-exposure group, while couples were classified into low-exposure, medium-exposure, or high-exposure groups. K-medoids clustering is an unsupervised learning algorithm that selects actual samples from the dataset as cluster centers, reducing sensitivity to noise and outliers (Yao *et al.*, 2021). The joint effects of trace metal element mixture on early embryological outcomes of IVF were assessed using quantile-based g-computation (QGC) and group-weighted quantile sum (groupWQS). Specifically, QGC is a multi-step approach that calculates marginal structural models to assess the effects of multiple exposures (Keil *et al.*, 2020). Additionally, groupWQS evaluates the joint effects of multiple exposure groups on the outcome by analyzing the weights and effect sizes of each exposure group, thereby assessing the contribution of each exposure group to the outcome variable (Wheeler *et al.*, 2021). The independent effects of trace metal elements on early embryological outcomes of IVF by ENR were assessed using the conventional generalized linear mixed model (GLMM). A Poisson distribution with a log link function was used to explore the relationships with the 2PN zygotes numbers, best-quality embryo numbers, and blastocyst numbers. A binomial distribution with a logit link function was applied to analyze the associations with the fertilization rates. Plasma trace element concentrations were incorporated in the GLMM as continuous variables and tertiles, with the lowest tertile as the reference. To improve the interpretation of the results, we converted the regression coefficients for count data into percent changes using the formula 100×(exp(β)−1). The details on the modeling constructions are presented in Supplementary File S1 and Supplementary Fig. S2.

According to existing literature, we identified potential confounding factors associated with levels of trace metal elements and embryo development (Wu *et al.*, 2020; Li *et al.*, 2022; Deng *et al.*, 2023). In the couple-based models, adjustments were made for sperm concentration (continuous), follicle-stimulating hormone (FSH) levels (continuous), ovulation stimulation protocols (categorical), season of sample collection (categorical), parity (continuous), age difference of each couple (continuous), male and female ages (continuous), body mass index (BMI, continuous), smoking status (categorical), and alcohol consumption (categorical) for both partners. In partner-specific models, we examined the relationship between trace metal concentrations and early embryological outcomes in IVF for each partner, while adjusting for each partner’s age, BMI, smoking status and alcohol consumption, sperm concentration (male), FSH levels (female), and sampling season. Furthermore, we further stratified the study population by parity (parity ≥ 1 or parity = 0) to evaluate the impact of previous pregnancies on the results in sensitivity analyses. All statistical analyses were performed using R version 4.3, with statistical significance defined at *P*-value <0.05.

## Results

### Descriptive statistics

The demographic characteristics of 1071 couples with 1369 cycles are presented in Table 1. The mean (SD) age of females and males was 32.60 (5.22) and 33.79 (5.89) years, respectively. Most females reported no alcohol consumption in the past 6 months at recruitment (80.1%), whereas most male partners did (62.7%). Compared to nulliparous couples, parous couples were older, had lower educational attainment, and male partners had a higher frequency of alcohol consumption.

**Table 1.** Population characteristics by parity status among couples (n = 1071).^a^

Characteristic	Overall (n = 1071)	Nulliparous (n = 683)	Parous (n = 388)	***P*-value** ^b^
**Female**				
Age (years)	32.60 (5.22)	30.65 (4.39)	36.04 (4.76)	<0.001*
BMI (kg/m^2^)				0.04*
<18.5	55 (5.1)	42 (6.1)	13 (3.4)	
18.5–23.9	639 (59.7)	407 (59.6)	232 (59.8)	
24–27.9	307 (28.7)	183 (26.8)	124 (32.0)	
≥28	70 (6.5)	51 (7.5)	19 (4.9)	
Education				<0.001*
Less than high school	529 (49.4)	258 (37.8)	271 (69.8)	
High school	180 (16.8)	129 (18.9)	51 (13.1)	
College and above	362 (33.8)	296 (43.3)	66 (17.0)	
Personal income (yuan/year)				0.22
<60 000	838 (78.2)	526 (77.0)	312 (80.4)	
≥60 000	233 (21.8)	47 (6.9)	76 (19.6)	
Active smoking				0.01*
Never	1012 (94.5)	636 (93.1)	376 (96.9)	
Former or current	59 (5.5)	47 (6.9)	12 (3.1)	
Passive smoking				0.74
Never	854 (79.7)	541 (79.2)	313 (80.7)	
Frequent	42 (3.9)	26 (3.8)	16 (4.1)	
Nearly every day	175 (16.3)	116 (17.0)	59 (15.2)	
Alcohol use				0.65
Never	858 (80.1)	553 (81.0)	305 (78.6)	
Frequent	205 (19.1)	125 (18.3)	80 (20.6)	
Nearly every day	8 (0.7)	5 (0.7)	3 (0.8)	
**Male**				
Age (years)	33.79 (5.89)	31.97 (5.10)	36.99 (5.84)	<0.001*
BMI (kg/m^2^)				0.35
<18.5	36 (3.4)	28 (4.1)	8 (2.1)	
18.5–24.9	510 (47.6)	325 (47.6)	185 (47.7)	
25–29.9	404 (37.7)	253 (37.0)	151 (38.9)	
≥30	121 (11.3)	77 (11.3)	44 (11.3)	
Education				<0.001*
Less than high school	453 (42.3)	235 (34.4)	218 (56.2)	
High school	220 (20.5)	144 (21.1)	76 (19.6)	
College and above	398 (37.2)	304 (44.5)	94 (24.2)	
Personal income (yuan/year)				0.08
<60 000	518 (48.4)	316 (46.3)	202 (52.1)	
≥60 000	553 (51.6)	367 (53.7)	186 (47.9)	
Active smoking				0.29
Never	452 (42.2)	297 (43.5)	155 (39.9)	
Former or Current	619 (57.8)	386 (56.5)	233 (60.1)	
Passive smoking				0.41
Never	699 (65.3)	453 (66.3)	246 (63.4)	
Frequent	85 (7.9)	49 (7.2)	36 (9.3)	
Nearly every day	287 (26.8)	181 (26.5)	106 (27.3)	
Alcohol use				<0.001*
Never	399 (37.3)	301 (44.1)	98 (25.3)	
Frequent	510 (47.6)	297 (43.5)	213 (54.9)	
Nearly every day	162 (15.1)	85 (12.4)	77 (19.8)	

* *P*-value <0.05.

a Values are presented as mean (SD) for continuous variables or as n (%) for categorical variables.

b *P*-values were calculated using chi-square tests for categorical variables and Student’s *t*-tests for continuous variables.

Among the 21 trace metal elements, except for beryllium (Be) which was detected in 52.8% of females and 54.3% of males, most trace metal elements were detected in >90% of couples (Supplementary Table S1). Concentrations of individual trace metal elements ranged from 0.05 to 3930.91 ng/ml in females and from 0.05 to 4339.15 ng/ml in males (Supplementary Table S1). Furthermore, Spearman correlation analysis indicated significant correlations among most trace metals within couples (Supplementary Fig. S3).

### Identification of trace metal elements associated with early embryological outcomes of IVF among male partners, female partners, and couples using ENR

In the ENR analysis, we identified trace metal elements from each partner associated with early embryological outcomes of IVF. Specifically, in males, among the 21 trace metal elements, we discovered that cobalt (Co), rubidium (Rb), strontium (Sr), silver (Ag), and thallium (Tl) were associated with the number of 2PN zygotes. Tin (Sn) and Ag were associated to the number of best-quality embryos, while aluminium (Al), nickel (Ni), gallium (Ga), Rb, cerium (Ce), Cu, Ag, and molybdenum (Mo) were associated with the fertilization rate. Additionally, 20 trace metal elements, excluding Ce, were associated with blastocyst numbers in males (Table 2 and Supplementary Table S2).

**Table 2.** Trace metal elements selected by ENR associated with early IVF embryological outcomes among male partners, females partners, and couples.

Elements	Early embryological outcomes of IVF											
	2PN zygote numbers			Fertilization rates			Best-quality embryo numbers			Blastocyst numbers		
	Male	Female	Couple	Male	Female	Couple	Male	Female	Couple	Male	Female	Couple
Be	—	Selected	—	—	—	—	—	Selected	—	Selected	Selected	Selected
Al	—	Selected	—	Selected	—	—	—	Selected	—	Selected	Selected	Selected
V	—	Selected	—	—	—	—	—	Selected	—	Selected	Selected	Selected
Li	—	Selected	—	—	—	—	—	Selected	—	Selected	Selected	Selected
Fe	—	Selected	—	—	—	—	—	Selected	—	Selected	Selected	Selected
Mn	—	Selected	—	—	Selected	—	—	Selected	—	Selected	Selected	Selected
Co	Selected	Selected	Selected	—	Selected	—	—	Selected	—	Selected	Selected	Selected
Ni	—	Selected	—	Selected	Selected	Selected	—	—	—	Selected	Selected	Selected
Ga	—	Selected	—	Selected	Selected	Selected	—	Selected	—	Selected	Selected	Selected
Rb	Selected	Selected	Selected	Selected	Selected	Selected	—	Selected	—	Selected	Selected	Selected
Sn	—	—	—	—	—	—	Selected	Selected	Selected	Selected	Selected	Selected
Sr	Selected	Selected	Selected	—	—	—	—	Selected	—	Selected	Selected	Selected
Ce	—	Selected	—	Selected	Selected	Selected	—	Selected	—	—	Selected	—
Cu	—	Selected	—	Selected	Selected	Selected	—	Selected	—	Selected	Selected	Selected
Ag	Selected	Selected	Selected	Selected	—	—	Selected	Selected	Selected	Selected	Selected	Selected
Cd	—	Selected	—	—	—	—	—	Selected	—	Selected	Selected	Selected
Ba	—	Selected	—	—	—	—	—	Selected	—	Selected	Selected	Selected
Se	—	Selected	—	—	—	—	—	Selected	—	Selected	Selected	Selected
Zn	—	Selected	—	—	—	—	—	Selected	—	Selected	Selected	Selected
Tl	Selected	Selected	Selected	—	—	—	—	Selected	—	Selected	Selected	Selected
Mo	—	Selected	—	Selected	Selected	Selected	—	Selected	—	Selected	Selected	Selected

‘—’ refers to trace metals in the ENR selection results that are not associated with the outcomes, i.e. trace metals that were not included in the couple-based and partner-specific analyses. ‘Selected’ refers to trace metals in the ENR selection results that are associated with the outcomes, i.e. trace metals that were included in both the couple-based and partner-specific analyses. ENR, elastic network regression; 2PN, two-pronuclear;.

In females, all trace metal elements except Sn were found to be associated with the number of 2PN zygotes, and all trace metal elements except Ni were found to be associated with the number of best-quality embryos. Manganese (Mn), Co, Ni, Ga, Rb, and Mo were identified as being associated with the fertilization rate. Furthermore, all 21 trace metal elements were found to be associated with blastocyst numbers (Table 2 and Supplementary Table S2).

In couples, six trace metals, including Co, Rb, Sr, Ag, and Tl, were found to be associated with the number of 2PN zygotes. Both Sn and Ag were associated with the number of best-quality embryos, while Ni, Ga, Rb, Ce, Cu, and Mo were associated with the fertilization rate. All elements, except Ce, were found to be associated with the blastocyst numbers in couples (Table 2 and Supplementary Table S2). The following analysis is based on trace metal elements identified by the ENR in this section.

### Effects of trace metal element mixture exposure patterns on early IVF embryological outcomes among couples, male partners, and female partners

Table 3 shows the associations between exposure patterns of trace metal element mixtures and early IVF embryo outcomes using K-medoids clustering in couples, males, and females. We observed that when couples were in the high-exposure group, the number of best-quality embryos decreased by 14.79% (95% confidence interval: −25.17%, −1.98%) and the number of blastocysts decreased by 25.17% (95% CI: −42.88%, −1.98%) compared to the low-exposure group (Table 3). Similarly, for male partners, high exposure resulted in a 15.63% (95% CI: −25.17%, −4.88%) decrease in best-quality embryos and a 22.12% (95% CI: −37.50%, −2.96%) decrease in blastocysts numbers compared to low-exposure. However, these associations were not found in female partners.

**Table 3.** Association of trace metal element mixtures exposure patterns and early IVF embryological outcomes among couples, females partners, and male partners using K-medoids clustering.

Exposure	Early embryological outcomes of IVF			
	**2PN zygotes numbers** ^a^ **percent changes (95% CI)**	**Fertilization rates** ^b^ **RR (95% CI)**	**Best-quality embryo numbers** ^a^ **percent changes (95% CI)**	**Blastocyst numbers** ^a^ **percent changes (95% CI)**
**Couple**				
Low-exposure	Reference	Reference	Reference	Reference
Medium-exposure	3.05 (−6.76,12.75)	1.02 (0.85,1.23)	−7.69 (−19.75,6.18)	10.52 (−13.06,41.91)
High-exposure	6.18 (−4.88,17.35)	1.01 (0.82,1.26)	−14.79 (−25.17,−1.98)*	−25.17 (−42.88,−1.98)*v
**Female partner**				
Low-exposure	Reference	Reference	Reference	Reference
High-exposure	4.08 (−3.92,12.75)	1.15 (0.98,1.37)	1.01 (−10.42,12.75)	3.05 (−16.47,27.12)
**Male partner**				
Low-exposure	Reference	Reference	Reference	Reference
High-exposure	−1.00 (−8.61,8.33)	1.04 (0.89,1.22)	−15.63 (−25.17,−4.88)*	−22.12 (−37.50,−2.96)*

a Data are presented as percent changes (95% CI).

b Data are presented as RR (95% CI). RR, relative risk; 2PN, two-pronuclear;

* *P*-value <0.05.

### Joint effects of trace metal mixture on early IVF embryological outcomes among couples, male partners, and female partners

**Figure 1.**
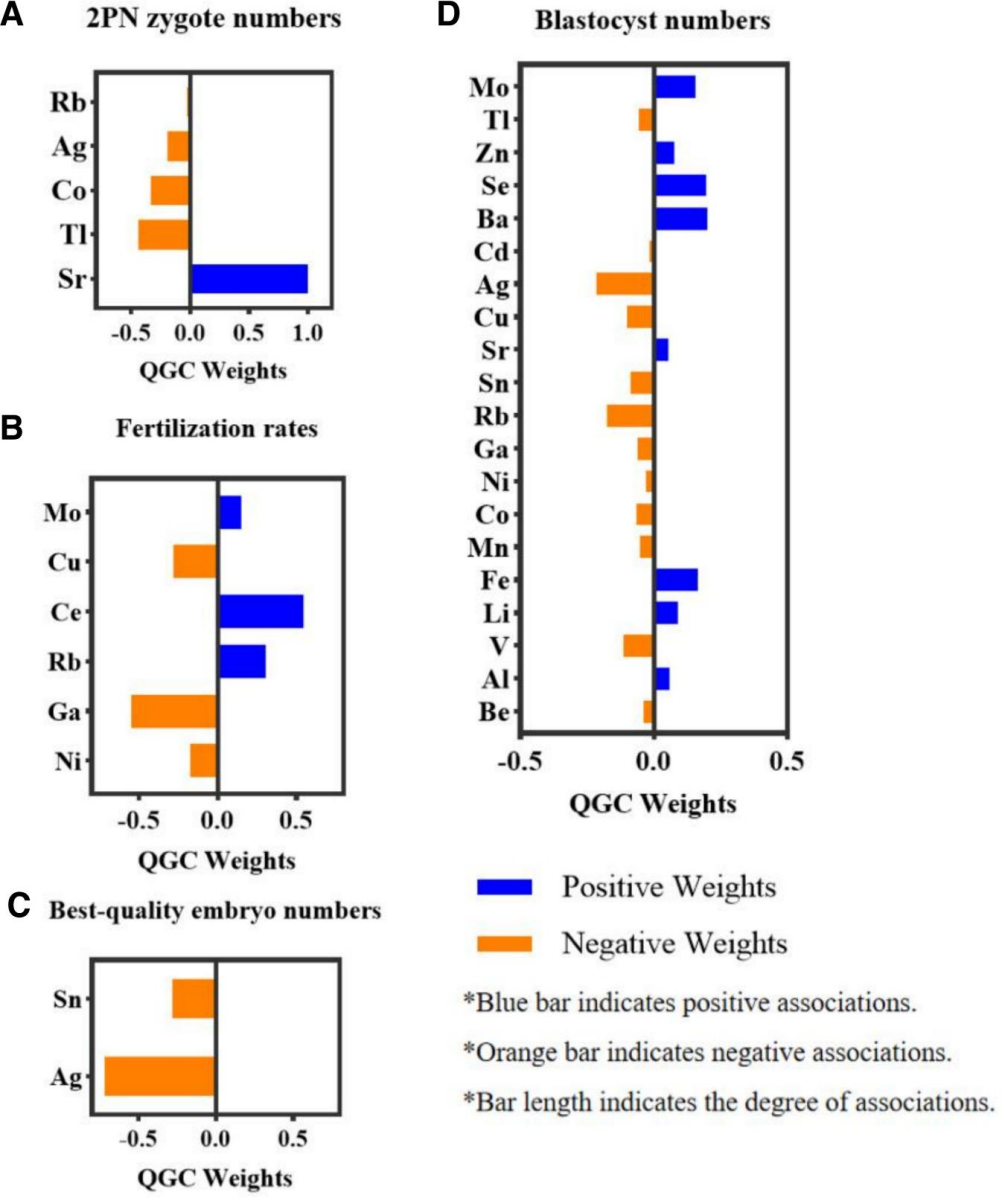
**Associations between ENR-selected trace metal element mixtures and early IVF embryological outcomes in couples.** The quantile-based g-computation (QGC) method was used for the mixture analysis. Estimates were adjusted for age, BMI, smoking, and drinking status from each partner, parity, sperm concentration, FSH levels, the age difference in the couple, ovulation stimulation protocol, and season of sampling. (**A**)–(**D**) represent the weights and directions of the associations between trace metal elements and the number of 2PN zygotes, fertilization rates, the number of best-quality embryos, and blastocyst numbers in the QGC analysis. ENR, elastic network regression; RR, relative risk; 2PN, two-pronuclear.

According to the trace metals identified by ENR to be associated with early embryological outcomes of IVF in couples, QGC determined their effect directions and weights of these trace metals on the outcomes. Among four trace metals identified by ENR to be associated with 2PN zygote numbers, Sr contributed the largest weight. For best-quality embryos and blastocyst numbers, Ag contributed the largest weight. Of the six trace metals associated with fertilization rate, Ce contributed the largest positive weight (Fig. 1). In the groupWQS analysis, per 1 ln unit increment in positive effect trace metal mixtures was associated with a 50.68% increase (95% CI: 19.72%, 91.55%) in 2PN zygote numbers and a 504.96% increase (95% CI: 309.60%, 784.63%) in blastocyst numbers. Conversely, a 1 ln unit increment in negative effect trace metal mixtures was associated with a 53.70% (95% CI: −70.18%, −28.82%) decrease in the 2PN zygote numbers, a 28.82% (95% CI: −39.35%, −16.47%) decrease in best-quality embryo numbers, and an 88.58% (95% CI: −92.86%, −81.91%) decrease in blastocyst numbers (Table 4).

**Table 4.** Association between grouped trace metal mixture and early embryological outcomes of IVF among couples, male partners, and female partners using groupWQS.

Outcomes	Positive effect group	Negative effect group
**Couple**		
2PN zygote numbers^a^	50.68 (19.72,91.55)*	−53.70 (−70.18,−28.82)*
Fertilization rates^b^	1.06 (1.04,1.08)*	0.94 (0.91,0.96)*
Best-quality embryo numbers^a^	—	−28.82 (−39.35,−16.47)*
Blastocyst numbers^a^	504.96 (309.60,784.63)*	−88.58 (−92.86,−81.91)*
**Male partner**		
2PN zygote numbers^a^	33.64 (4.08,71.60)*	−30.93 (−55.07,−5.82)*
Fertilization rates^b^	1.08 (1.05,1.12)*	0.94 (0.91,0.96)*
Best-quality embryo numbers^a^	—	−26.66 (−36.87,−13.93)*
Blastocyst numbers^a^	420.70 (249.03,676.79)*	−86.73 (−91.71,−78.99)*
**Female partner**		
2PN zygote numbers^a^	405.31 (200.42,749.94)*	−87.00 (−92.79,−76.54)*
Fertilization rates^b^	1.07 (1.05,1.09)*	0.95 (0.92,0.97)*
Best-quality embryo numbers^a^	235.35 (133.96,380.66)*	−77.91 (−85.19,−67.04)*
Blastocyst numbers^a^	535.98 (330.60,829.99)*	−87.25 (−92.11,−79.40)*

a Data are presented as percent changes (95% CI).

b Data are presented as RR (95% CI). groupWQS, group-weighted quantile sum regression; The ‘—’ indicates the absence of trace metal elements in this effect group.

* *P*-value <0.05.

In males, the effect directions and weights of trace metal elements determined by QGC were generally consistent with the outcomes observed in couples (Supplementary Fig. S4). However, in contrast, Ga had the largest negative weight in fertilization rates. In the groupWQS analysis, the results for males were generally consistent with those for couples (Table 4).

In females, among the effect direction and weight of trace metal elements associated with outcomes, Ag contributed the largest negative weight for both blastocyst numbers and best-quality embryo numbers (Supplementary Fig. S5). For best-quality embryo numbers, Fe contributed the largest positive weight, while Mn had the largest positive weight for fertilization rates. Notably, the groupWQS results for females remained consistent with those for couples, although the positive effects were 10 times greater 2PN zygote numbers and there was a large positive effect for best-quality embryo numbers (Table 4).

### Independent effects of individual trace metal element on early IVF embryological outcomes among couples, male partners, and female partners

**Figure 2.**
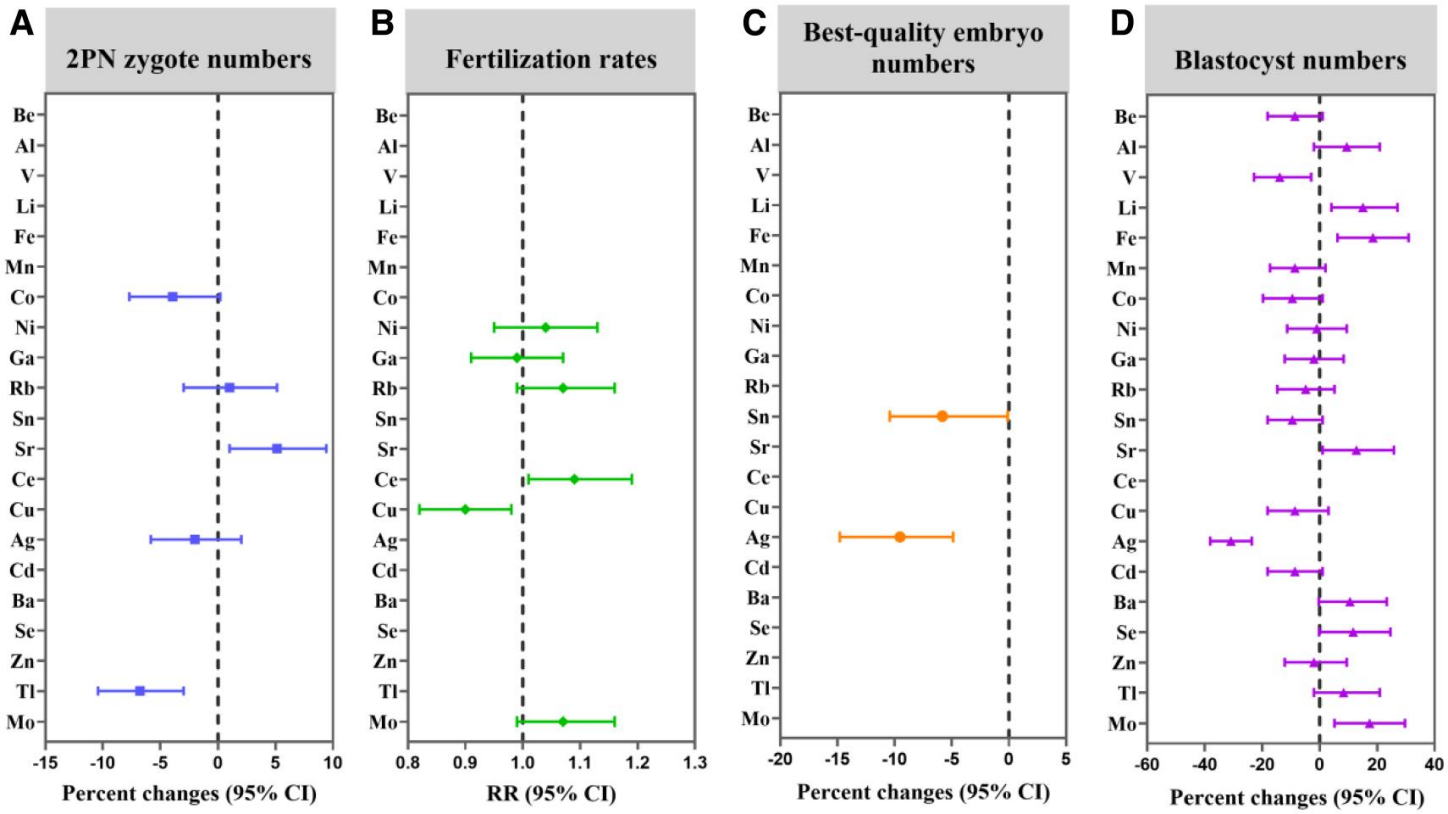
**Associations between ENR-selected individual trace metal elements and early IVF embryological outcomes among 1071 couples with 1369 IVF cycles.** The analyses were conducted using generalized linear mixed models with random intercepts. Estimates were adjusted for age, BMI, smoking, and drinking status from each partner, parity, sperm concentration, FSH levels, the age difference in the couple, ovulation stimulation protocol, and season of sampling. For (**A**), (**C**), and (**D**), a Poisson distribution and log link function were applied, with data presented as percent changes (95% CI). For (**B**), a binomial distribution and logit link function were utilized, with data presented as RR (95% CI). ENR, elastic network regression; RR, relative risk; 2PN, two-pronuclear.

Table 5 and Fig. 2 show the associations between individual trace metal elements and early IVF embryological outcomes in couples. In couples, per 1 ln unit increment in Sr was associated with a 5.13% increase in 2PN zygote numbers. Compared with the couples in the lowest tertile, couples in the highest tertile of Sr showed a 12.75% increase in 2PN zygote numbers. Conversely, per 1 ln unit increment in Tl was associated with a 6.76% decrease in 2PN zygote numbers and a 13.06% decrease in the highest tertile compared to the lowest tertile. For best-quality embryos, per 1 ln unit increment in Sn and Ag was associated with 5.82% and 9.52% decreases in best-quality embryos, respectively. Compared with the couples in the lowest tertile, those in the highest tertile of Sn and Ag showed an 18.94% and 21.34% decrease in best-quality embryos, respectively. For fertilization rate, Ce showed a positive association, while Cu showed a negative association. For blastocyst numbers, per 1 ln unit increment in lithium (Li), Fe, and Mo were associated with 15.03%, 18.53%, and 17.35% increases in blastocyst numbers, respectively. Compared with the couples in the lowest tertile, those in the highest tertile of Li, Fe, and Mo showed a 32.31%, 44.77%, and 64.87% increase in blastocyst numbers. In contrast, per 1 ln unit increment in Ag was associated with a 17.35% reduction in blastocyst numbers and a 57.68% decrease in the highest tertile compared to the lowest tertile. Stratification by parity did not change the significant associations between trace metal elements and early IVF embryological outcomes (all *P* for interactions >0.05) (Supplementary Tables S3–S6, Supplementary Fig. S6).

**Table 5.** Associations between ENR selected individual trace metal element with early IVF embryological outcomes among 1071 couples with 1369 IVF cycles.

Early embryological outcomes of IVF	Element	**Continuous** ^c^	**Tertiles** ^d^		
			T1	T2	T3
2PN zygote numbers^a^	Co	−3.92 (−7.69,0.20)	Reference	−4.88 (−4.88,4.08)	−10.42 (−18.94,−1.00)*
	Rb	1.01 (−2.96,5.13)	Reference	−5.82 (−14.79,3.05)	−1.00 (−10.42,9.42)
	Sr	5.13 (1.01,9.42)*	Reference	8.33 (−1.00,8.33)	12.75 (2.02,24.61)*
	Ag	−1.98 (−5.82,2.02)	Reference	−6.76 (−15.63,3.05)	−4.88 (−13.93,5.13)
	Tl	−6.76 (−10.42,−2.96)*	Reference	−7.69 (−16.47,1.01)	−13.06 (−20.55,−3.92)*
Fertilization rates^b^	Ni	1.04 (0.95,1.13)	Reference	0.96 (0.79,1.16)	0.98 (0.80,1.21)
	Ga	0.99 (0.91,1.07)	Reference	0.81 (0.66,0.99)*	0.84 (0.68,1.02)
	Rb	1.07 (0.99,1.16)	Reference	1.04 (0.85,1.26)	1.11 (0.90,1.35)
	Ce	1.09 (1.01,1.19)*	Reference	0.84 (0.69,1.02)	1.19 (0.98,1.46)
	Cu	0.90 (0.82,0.98)*	Reference	0.90 (0.73,1.09)	0.82 (0.66,1.02)
	Mo	1.07 (0.99,1.16)	Reference	1.19 (0.97,1.43)	1.05 (0.87,1.28)
Best-quality embryo numbers^a^	Sn	−5.82 (−10.42,−0.10)*	Reference	−18.94 (−29.53,−7.69)*	−11.31 (−22.12,1.01)
	Ag	−9.52 (−14.79,−4.88)*	Reference	−17.30 (−27.39,−5.82)*	−21.34 (−31.61,−10.42)*
Blastocyst numbers^a^	Be	−8.61 (−18.13,1.01)	Reference	3.05 (−21.34,33.64)	−15.63 (−34.95,9.42)
	Al	9.42 (−1.98,20.92)	Reference	22.14 (−5.82,56.83)	22.14 (−5.82,58.41)
	V	−13.93 (−22.89,−2.96)*	Reference	−16.47 (−35.60,9.42)	−12.19 (−32.97,15.03)
	Li	15.03 (4.08,27.12)*	Reference	3.05 (−20.55,33.64)	32.31 (2.02,71.60)*
	Fe	18.53 (6.18,31.00)*	Reference	27.12 (−1.98,64.87)	44.77 (11.63,87.76)*
	Mn	−8.61 (−17.30,2.02)	Reference	−3.92 (−25.92,24.61)	3.05 (−21.34,33.64)
	Co	−9.52 (−19.75,1.01)	Reference	−21.34 (−38.74,2.02)	−25.92 (−43.45,−3.92)*
	Ni	−1.00 (−11.31,9.42)	Reference	27.12 (−1.00,63.23)	−1.98 (−25.17,28.40)
	Ga	−1.98 (−12.19,8.33)	Reference	−17.30 (−36.24,7.25)	1.01 (−22.89,31.00)
	Rb	−4.88 (−14.79,5.13)	Reference	−13.93 (−33.63,11.63)	−22.89 (−40.55,1.01)
	Sn	−9.52 (−18.13,1.01)	Reference	−31.61 (−47.27,−12.19)*	−26.66 (−44.01,−4.88)*
	Sr	12.75 (1.01,25.86)*	Reference	10.52 (−14.79,41.91)	20.92 (−7.69,56.83)
	Cu	−8.61 (−18.13,3.05)	Reference	6.18 (−18.94,37.71)	−14.79 (−35.60,12.75)
	Ag	−30.93 (−38.12,−23.66)*	Reference	−22.89 (−39.95,−1.98)*	−57.68 (−67.37,−45.12)*
	Cd	−8.61 (−18.13,1.01)	Reference	−10.42 (−30.23,15.03)	−27.39 (−44.01,−5.82)*
	Ba	10.52 (−0.30,23.37)	Reference	13.88 (−12.19,46.23)	6.18 (−18.13,37.71)
	Se	11.63 (−0.20,24.61)	Reference	34.99 (5.13,75.07)*	44.77 (10.52,87.76)*
	Zn	−1.98 (−12.19,9.42)	Reference	1.01 (−22.12,31.00)	27.12 (−1.98,64.87)
	Tl	8.33 (−1.98,20.92)	Reference	−15.63 (−34.95,9.42)*	−15.63 (−34.95,9.42)*
	Mo	17.35 (5.13,29.69)*	Reference	69.89 (31.00,120.34)*	64.87 (27.12,113.83)*

a Data are presented as percent changes (95% CI).

b Data are presented as RR (95% CI).

c Elements were modeled as continuous variables in the multivariate generalized linear mixed model.

d Elements were modeled as categorical variables (tertiles) in the multivariate generalized linear mixed model. Notes: ENR, elastic network regression; RR, relative risk; 2PN, two-pronuclear;

* *P*-value <0.05.

In male and female partners, the results were generally consistent with the GLMM analysis from couples (Supplementary Tables S7 and S8, Supplementary Figs S7 and S8). The findings that differ from the couples' results are that Rb in males showed the significant positive association with fertilization rates, while Be, Ga, Sn, and Cu in males were negatively associated with blastocyst numbers. Additionally, plasma Fe in females was positively associated with all four early IVF embryological outcomes. When modeled using tertiles, these associations generally persisted (Supplementary Figs S7 and S8).

## Discussion

In this prospective couple-based cohort study, we explored the association between 21 trace metal elements and early embryological outcomes of IVF using multi-stage statistical analysis strategy. The study revealed significant associations between trace metals and early IVF outcomes at the couple and individual partner levels.

First, the detection rates of most trace metal elements in our study exhibited high detection rates, except for Be, and the concentrations of elements were mostly within the reference ranges reported in previous studies (Gonzalez-Martin *et al.*, 2023; Palomar *et al.*, 2023). Additionally, our findings revealed correlations in trace metal element concentrations in plasma within couples, which further highlights the significance of studying co-exposure to these elements in couples in relation to fertility.

Moreover, it is well known that exposure to trace metal elements is not limited to a single element, as trace metals exist in a variety of forms and interact with each other. In light of this complexity, we employed several mixture analysis approaches to explore the combined effects of metal mixtures on reproductive outcomes, in order to better reflect the actual exposure scenarios faced by individuals. After selecting the metal elements associated with reproductive outcomes using ENR, key elements closely associated with the outcomes were precisely identified, effectively minimizing the influence of irrelevant trace elements. The exposure patterns of metal mixtures in couples were significantly associated with early IVF embryological outcomes. Using the K-medoids clustering method, couples were categorized into low-, medium-, and high-exposure groups. Compared to the low-exposure group, the high-exposure group showed a 14.79% decrease in high-quality embryos and a 25.17% decrease in blastocyst numbers. Furthermore, the analysis based on metal mixtures in couples showed that harmful metal mixtures, particularly those containing Ag, were negatively associated with key embryological outcomes, including a decrease in the number of 2PN zygotes and blastocysts. In contrast, beneficial metal mixtures, especially those containing Sr and Mo, were positively correlated with improved early IVF embryological outcomes.

Finally, we focused our analysis on the impact of individual trace metal elements on embryological outcomes, allowing for a more detailed investigation into the specific effects of each metal. In addition, the results of GLMM analysis for couples and GLMM analysis for both partners were generally consistent. Specifically, for females, Fe and Sr were positively associated with both 2PN zygote numbers and blastocyst numbers while Se and Mo showed positive associations with blastocyst numbers. For males, the results indicated that Sr was positively associated with 2PN zygote numbers while Mo showed a positive association with blastocyst numbers. Ag was negatively associated with all four early IVF embryological outcomes. Additionally, Cu showed a negative relationship with both fertilization rates and blastocyst numbers.

These findings highlight the complex impact of both individual trace metals and their mixtures on early IVF embryological outcomes, suggesting that exposure patterns vary between partners, and that specific metals may either enhance or impair embryological development. The observed variability in exposure patterns between partners and couples suggests that the effects of trace metals on embryonic development are not uniform, and may differ based on gender, genetic factors, or pre-existing health conditions (Wang *et al.*, 2017; Gonzalez-Martin *et al.*, 2023; Shen *et al.*, 2023). Future research may further explore the associations of trace metal elements in follicular fluid with ovarian response among females, as well as the associations of trace metal elements in seminal plasma with semen parameters among males, to comprehensively assess the impact of trace metals on reproductive health. The combination of these elements in mixtures further complicates their effects, as synergistic or antagonistic interactions may occur, resulting in outcomes that cannot be predicted by the impact of individual metals alone. The interactions between metal elements highlight the need for more detailed research on environmental exposures in the field of reproductive medicine. Specifically, future research should explore the potential mechanisms through which interactions between metal elements affect embryonic development, in order to gain a more comprehensive understanding of how environmental factors influence the development of embryos.

Furthermore, whether exposure to trace metal elements affects early embryological outcomes of IVF remains under debate. In our study, we observed that Cd has a significant harmful effect on blastocyst development during IVF treatment, whereas Se and Sr have substantial positive effects. A prospective cohort study of 1184 females in China reported a negative association between Cd and best-quality embryo rates, and a positive association between Se and mature oocyte rates (MII), which is consistent with our results (Li *et al.*, 2022). Moreover, a randomized controlled trial demonstrated that Sr enhances fertilization rates by activating oocytes, which supports our findings (Fawzy *et al.*, 2018). However, several prospective studies with small-scale American females found that the association between Fe and IVF outcomes was not significant (Gonzalez-Martin *et al.*, 2023, 2024). Notably, they also observed a positive effect of Cu on IVF embryological outcomes, which is inconsistent with our results. The differences in research results may be due to variations in population characteristics and sample sizes. Furthermore, it has been reported that the essential trace element Mo in urine is harmful to IVF outcomes, which is inconsistent with our results, possibly due to differences in the biological samples tested. Additionally, research on Ag and embryo development is limited. An animal study found that metal ions such as Cu and Ag cause developmental abnormalities in zebrafish embryos, which may provide further insights into their effects on embryo development (Tai *et al.*, 2019).

The toxic effects of metals on reproductive health have been widely confirmed. Embryonic development is a highly plastic process that is relatively sensitive to environmental factors. Exposure to metals in the environment may influence embryonic development. One potential mechanism is that metals affect the expression of Dvr1, resulting in asymmetrical embryonic development (Li *et al.*, 2012; Zargari *et al.*, 2022; Liu *et al.*, 2023). Additionally, another possible explanation was that metal toxicity may have led to oxidative damage in the reproductive system. Toxic metals could have disrupted reproductive physiological processes by influencing reactive oxygen species, which could have affected oocyte maturation, implantation, and blastocyst formation (Lu *et al.*, 2018). Furthermore, animal studies have confirmed that Ag nanoparticles exhibit toxic effects on early embryonic development, which may be attributed to oxidative stress (Lee *et al.*, 2007; Ahamed *et al.*, 2010). Several studies have found that Sr plays an important role in regulating male reproductive function. It not only increases the number of sperm in the epididymis but also enhances testosterone levels (Feng *et al.*, 2007; Huang *et al.*, 2023). In addition, previous studies have indicated that Mo, as an essential trace metal, could improve the quantity of sperm and oocytes at low doses. Overall, metals have bidirectional effects on embryonic development and reproductive health, and the underlying mechanisms need further investigation to be fully understood.

Our study has several strengths. First, we measured various trace metal element concentrations in couples in a multi-center prospective cohort, which allowed us to comprehensively investigate how these elements impact early embryological outcomes in IVF. Second, the couple-based design and large sample size enabled us to assess the effects of trace metal elements contributed by both partners with considerable precision. It is worth noting that the use of metal element levels in blood, especially plasma, as biomarkers can better reflect the accumulation of metals and the long-term exposure of individuals than the level of metal elements in urine. Moreover, we utilized multiple multipollutant models (i.e. ENR, K-medoids, QGC, and groupWQS) alongside conventional single pollutant models (i.e. GLMM) to examine the joint and individual effects of 21 trace metal elements, which helped to identify exposures with strong associations.

Nevertheless, this study has several limitations. First, plasma trace metal concentrations were assessed at a single time point, potentially overlooking temporal variations in exposure. Additionally, only total elemental concentrations were measured, without distinguishing between chemical species or valence states. This is critical, as toxicity varies by form, for instance, soluble barium compounds pose greater health risks than insoluble ones (Al Osman *et al.*, 2019). Second, our analysis focused solely on fresh embryo transfer cycles, limiting applicability to other ART cycles. Unmeasured confounders, such as psychological stress or lifestyle factors, were also not accounted for. Finally, the study population comprised couples undergoing IVF/ART, necessitating caution in extrapolating findings to the general population. However, this focus holds clinical relevance, as ART populations may exhibit heightened susceptibility to environmental toxicants due to underlying fertility challenges. With the global rise in ART utilization, our findings provide actionable insights into optimizing treatment success by addressing modifiable environmental factors. Future studies should integrate longitudinal exposure assessments, chemical speciation analyses, and broader population cohorts to strengthen causal inferences.

## Conclusions

Our study revealed that exposure to trace metal elements significantly impacts early IVF embryological outcomes in couples and both partners. High exposure to mixtures of trace metal elements in couples and male partners was associated with decreased numbers of best-quality embryos and blastocysts. By QGC and groupWQS, we identified both harmful and beneficial metal mixtures that influence reproductive success. Additionally, specific trace metals such as Fe, Sr, and Mo were positively associated with embryological outcomes, while metals like Ag and Tl had adverse effects. These findings emphasize the importance of considering trace metal exposure as a modifiable factor in IVF success. These findings highlight the importance of implementing preconception trace element screening and targeted trace element interventions for couples planning to conceive, as a strategy to optimize reproductive health and IVF outcomes.

## Supplementary Material

hoaf034_Supplementary_Data

## Data Availability

The data underlying this article will be shared on reasonable request to the corresponding author.

## References

[hoaf034-B1] Ahamed M , AlSalhi MS, Siddiqui MKJ. Silver nanoparticle applications and human health. Clin Chim Acta 2010;411:1841–1848. 20719239 10.1016/j.cca.2010.08.016

[hoaf034-B2] Ajduk A , Zernicka-Goetz M. Quality control of embryo development. Mol Asp Med 2013;34:903–918. 10.1016/j.mam.2013.03.001 23563243

[hoaf034-B3] Al Osman M , Yang F, Massey IY. Exposure routes and health effects of heavy metals on children. Biometals 2019;32:563–573. 30941546 10.1007/s10534-019-00193-5

[hoaf034-B4] Allouche-Fitoussi D , Breitbart H. The role of zinc in male fertility. Int J Mol Sci 2020;21:7796. 33096823 10.3390/ijms21207796 PMC7589359

[hoaf034-B5] Chu T , Wang D, Yu T, Zhai J. Effects of seasonal variations and meteorological factors on IVF pregnancy outcomes: a cohort study from Henan Province, China. Reprod Biol Endocrinol 2022;20:113. 35933344 10.1186/s12958-022-00986-3 PMC9356437

[hoaf034-B6] Deng YL , Liu C, Yuan XQ, Luo Q, Miao Y, Chen PP, Cui FP, Zhang M, Zeng JY, Shi T et al Associations between urinary concentrations of disinfection byproducts and in vitro fertilization outcomes: a prospective cohort study in China. Environ Health Perspect 2023;131:97003. 37671782 10.1289/EHP12447 PMC10481678

[hoaf034-B7] Dutta S , Gorain B, Choudhury H, Roychoudhury S, Sengupta P. Environmental and occupational exposure of metals and female reproductive health. Environ Sci Pollut Res Int 2022;29:62067–62092. 34558053 10.1007/s11356-021-16581-9

[hoaf034-B8] Fawzy M , Emad M, Mahran A, Sabry M, Fetih AN, Abdelghafar H, Rasheed S. Artificial oocyte activation with SrCl2 or calcimycin after ICSI improves clinical and embryological outcomes compared with ICSI alone: results of a randomized clinical trial. Hum Reprod 2018;33:1636–1644. 30099496 10.1093/humrep/dey258

[hoaf034-B9] Feng R , Chen B. Environmental risks and infertility in China. Science 2024;383:267–268. 10.1126/science.adn3214 38236987

[hoaf034-B10] Feng Y , Zhang Q, Dai DZ, Ying HJ, Dai Y. Strontium fructose 1,6-diphosphate rescues adenine-induced male hypogonadism and upregulates the testicular endothelin-1 system. Clin Exp Pharmacol Physiol 2007;34:1131–1137. 17880366 10.1111/j.1440-1681.2007.04693.x

[hoaf034-B11] Gonzalez-Martin R , Palomar A, Perez-Deben S, Salsano S, Quiñonero A, Caracena L, Fernandez-Saavedra R, Fernandez-Martinez R, Conde-Vilda E, Quejido AJ et al Higher concentrations of essential trace elements in women undergoing IVF may be associated with poor reproductive outcomes following single euploid embryo transfer. Cells 2024;13:839. 38786061 10.3390/cells13100839 PMC11119764

[hoaf034-B12] Gonzalez-Martin R , Palomar A, Quiñonero A, Pellicer N, Fernandez-Saavedra R, Conde-Vilda E, Quejido AJ, Whitehead C, Scott RT Jr, Dominguez F. The impact of essential trace elements on ovarian response and reproductive outcomes following single euploid embryo transfer. Int J Mol Sci 2023;24:10968. 37446146 10.3390/ijms241310968 PMC10341631

[hoaf034-B13] Huang X , Gao Y, Zhang Y, Wang J, Zheng N. Strontium chloride improves reproductive function and alters gut microbiota in male rats. Int J Mol Sci 2023;24:13922. 37762223 10.3390/ijms241813922 PMC10531462

[hoaf034-B14] Jin HX , Guo YH, Song WY, Li G, Liu Y, Shi SL. Effect of ambient air pollutants on in vitro fertilization-embryo transfer pregnancy outcome in Zhengzhou, China. Environ Toxicol Pharmacol 2022;90:103807. 34990867 10.1016/j.etap.2021.103807

[hoaf034-B15] Keil AP , Buckley JP, O’Brien KM, Ferguson KK, Zhao S, White AJ. A quantile-based g-computation approach to addressing the effects of exposure mixtures. Environ Health Perspect 2020;128:47004. 32255670 10.1289/EHP5838 PMC7228100

[hoaf034-B16] Kim K , Bloom MS, Kruger PC, Parsons PJ, Arnason JG, Byun Y, Goins S, Fujimoto VY. Toxic metals in seminal plasma and in vitro fertilization (IVF) outcomes. Environ Res 2014;133:334–337. 25019469 10.1016/j.envres.2014.06.014

[hoaf034-B17] Lee KJ , Nallathamby PD, Browning LM, Osgood CJ, Xu XH. In vivo imaging of transport and biocompatibility of single silver nanoparticles in early development of zebrafish embryos. ACS Nano 2007;1:133–143. 19122772 10.1021/nn700048y PMC2613370

[hoaf034-B18] Li D , Liang C, Cao Y, Zhu D, Shen L, Zhang Z, Jiang T, Zhang Z, Zong K, Liu Y et al The associations of serum metals concentrations with the intermediate and pregnancy outcomes in women undergoing in vitro fertilization (IVF). Ecotoxicol Environ Saf 2022;233:113309. 35183814 10.1016/j.ecoenv.2022.113309

[hoaf034-B19] Li X , Ma Y, Li D, Gao X, Li P, Bai N, Luo M, Tan X, Lu C, Ma X. Arsenic impairs embryo development via down-regulating Dvr1 expression in zebrafish. Toxicol Lett 2012;212:161–168. 22613031 10.1016/j.toxlet.2012.05.011

[hoaf034-B20] Liu M , Wu K, Wu Y. The emerging role of ferroptosis in female reproductive disorders. Biomed Pharmacother 2023;166:115415. 37660655 10.1016/j.biopha.2023.115415

[hoaf034-B21] Liu X , Luo K, Zhang J, Yu H, Chen D. Exposure of preconception couples to legacy and emerging per- and polyfluoroalkyl substances: variations within and between couples. Environ Sci Technol 2022;56:6172–6181. 35016501 10.1021/acs.est.1c07422

[hoaf034-B22] Lu J , Wang Z, Cao J, Chen Y, Dong Y. A novel and compact review on the role of oxidative stress in female reproduction. Reprod Biol Endocrinol 2018;16:80. 30126412 10.1186/s12958-018-0391-5 PMC6102891

[hoaf034-B23] Palomar A , Gonzalez-Martin R, Quiñonero A, Pellicer N, Fernandez-Saavedra R, Rucandio I, Fernandez-Martinez R, Conde-Vilda E, Quejido AJ, Zuckerman C et al Bioaccumulation of non-essential trace elements detected in women’s follicular fluid, urine, and plasma is associated with poor reproductive outcomes following single euploid embryo transfer: a pilot study. Int J Mol Sci 2023;24:13147. 37685954 10.3390/ijms241713147 PMC10487767

[hoaf034-B24] Penzias AS. Recurrent IVF failure: other factors. Fertil Steril 2012;97:1033–1038. 22464759 10.1016/j.fertnstert.2012.03.017

[hoaf034-B25] Polyzos NP , Drakopoulos P, Parra J, Pellicer A, Santos-Ribeiro S, Tournaye H, Bosch E, Garcia-Velasco J. Cumulative live birth rates according to the number of oocytes retrieved after the first ovarian stimulation for in vitro fertilization/intracytoplasmic sperm injection: a multicenter multinational analysis including ∼15,000 women. Fertil Steril 2018;110:661–670.e1. 30196963 10.1016/j.fertnstert.2018.04.039

[hoaf034-B26] Pons MC , Carrasco B, Rives N, Delgado A, Martínez-Moro A, Martínez-Granados L, Rodriguez I, Cairó O, Cuevas-Saiz I; SIG Embryology of ASEBIR. Predicting the likelihood of live birth: an objective and user-friendly blastocyst grading system. Reprod Biomed Online 2023;47:103243. 37473718 10.1016/j.rbmo.2023.05.015

[hoaf034-B27] Shen L , Liang C, Li D, Zhang Z, Wang X, Jiang T, Su X, Yin T, Zou W, Wang X et al The association between exposure to multiple toxic metals and the risk of endometriosis: evidence from the results of blood and follicular fluid. Sci Total Environ 2023;855:158882. 36155031 10.1016/j.scitotenv.2022.158882

[hoaf034-B28] Shi X , Ren MQ, Wang LT, Chan CPS, Chan DYL, Quan S, Li TC. Blood metal/metalloid concentration of male subjects undergoing IVF/ICSI treatment outcomes: a prospective cohort study. J Trace Elem Med Biol 2023;78:127196. 37209528 10.1016/j.jtemb.2023.127196

[hoaf034-B29] Sunderam S , Kissin DM, Zhang Y, Jewett A, Boulet SL, Warner L, Kroelinger CD, Barfield WD. Assisted Reproductive Technology Surveillance—United States, 2018. MMWR Surveill Summ 2022;71:1–19. 10.15585/mmwr.ss7104a1 PMC8865855 35176012

[hoaf034-B30] Tai Z , Guan P, Wang Z, Li L, Zhang T, Li G, Liu JX. Common responses of fish embryos to metals: an integrated analysis of transcriptomes and methylomes in zebrafish embryos under the stress of copper ions or silver nanoparticles. Metallomics 2019;11:1452–1464. 31468037 10.1039/c9mt00125e

[hoaf034-B31] Tian T , Wang Z, Liu F, Fu Y, Kong F, Wang Y, Li Q, Long X, Qiao J. Exposure to heavy metallic and trace essential elements and risk of diminished ovarian reserve in reproductive age women: a case-control study. J Hazard Mater 2024;470:134206. 38583203 10.1016/j.jhazmat.2024.134206

[hoaf034-B32] Wang L , Liang R, Zhang G, Ren M, Long M, Na J, Li Z, Wang B, Zhuang L, Lu Q. Serum zinc concentration and risk of adverse outcomes to in vitro fertilization and embryo transfer: a prospective cohort study in northern China. Sci Total Environ 2021;792:148405. 34153763 10.1016/j.scitotenv.2021.148405

[hoaf034-B33] Wang X , Ding N, Harlow SD, Randolph JF Jr, Mukherjee B, Gold EB, Park SK. Exposure to heavy metals and hormone levels in midlife women: the Study of Women’s Health Across the Nation (SWAN). Environ Pollut 2023;317:120740. 36436662 10.1016/j.envpol.2022.120740 PMC9897061

[hoaf034-B34] Wang YX , Wang P, Feng W, Liu C, Yang P, Chen YJ, Sun L, Sun Y, Yue J, Gu LJ et al Relationships between seminal plasma metals/metalloids and semen quality, sperm apoptosis and DNA integrity. Environ Pollut 2017;224:224–234. 28274591 10.1016/j.envpol.2017.01.083

[hoaf034-B35] Wheeler DC , Rustom S, Carli M, Whitehead TP, Ward MH, Metayer C. Assessment of grouped weighted quantile sum regression for modeling chemical mixtures and cancer risk. Int J Environ Res Public Health 2021;18:504. 33435473 10.3390/ijerph18020504 PMC7827322

[hoaf034-B36] Wu S , Wang M, Deng Y, Qiu J, Zhang X, Tan J. Associations of toxic and essential trace elements in serum, follicular fluid, and seminal plasma with in vitro fertilization outcomes. Ecotoxicol Environ Saf 2020;204:110965. 32798747 10.1016/j.ecoenv.2020.110965

[hoaf034-B37] Yao X , Steven Xu X, Yang Y, Zhu Z, Zhu Z, Tao F, Yuan M. Stratification of population in NHANES 2009–2014 based on exposure pattern of lead, cadmium, mercury, and arsenic and their association with cardiovascular, renal and respiratory outcomes. Environ Int 2021;149:106410. 33548850 10.1016/j.envint.2021.106410

[hoaf034-B38] Zargari F , Rahaman MS, KazemPour R, Hajirostamlou M. Arsenic, oxidative stress and reproductive system. J Xenobiot 2022;12:214–222. 35893266 10.3390/jox12030016 PMC9326564

