# Supplementary material for: Association between trace metal element concentrations in human blood plasma and early MAR embryological outcomes: a couple-based prospective cohort study
Source: Hum Reprod Open. 2025 Jun 10;2025(3):hoaf034. doi: 10.1093/hropen/hoaf034 (PMC12311266; doi:10.1093/hropen/hoaf034)
Supplement: hoaf034_Supplementary_Data [file hoaf034_supplementary_data.zip › HRO-24-0359-R2_Supplementary Information.docx]

**Supplementary Information**

**Association Between Trace metal element concentrations in human blood plasma and** **early MAR embryological outcomes:** **A Couple-based Prospective Cohort Study**

**Supplementary File S1.** Model construction.

**Supplementary Figures**

Supplementary Figure S1. The flowchart of study participants included in the present study.

Supplementary Figure S2. Detailed information on statistical modelling.

Supplementary Figure S3. Spearman correlation matrix of trace metal elements within each couple.

Supplementary Figure S4. Associations between ENR selected trace metal element mixtures and early embryological outcomes of IVF in males (QGC).

Supplementary Figure S5. Associations between ENR selected trace metal element mixtures and early embryological outcomes of IVF in females (QGC).

Supplementary Figure S6. Associations between ENR selected individual trace metal elements with early IVF embryological outcomes was stratified by parity.

Supplementary Figure S7. Associations between ENR selected individual trace metal element from with early IVF embryological outcomes in males.

Supplementary Figure S8. Associations between ENR selected individual trace metal element from with early IVF embryological outcomes in females.

**Supplementary Table****s**

Supplementary Table S1. Detection and distribution of plasma trace metal element concentrations in couples (N=1,071).

Supplementary Table S2. Associations between trace metal elements and early embryological outcomes of IVF in couple-based ENR analyses.

Supplementary Table S3. Associations between ENR selected individual trace metal element and blastocyst numbers in nulliparous and parous couples.

Supplementary Table S4. Associations between ENR selected individual trace metal element and fertilization rates in nulliparous and parous couples.

Supplementary Table S5. Associations between ENR selected individual trace metal element and 2PN numbers in nulliparous and parous couples.

Supplementary Table S6. Associations between ENR selected individual trace metal element and best-quality embryo numbers in nulliparous and parous couples.

Supplementary Table S7. Associations between ENR selected individual trace metal element with early IVF embryological outcomes in males.

Supplementary Table S8. Associations between ENR selected individual trace metal element with early IVF embryological outcomes in females.

‘

**SUPPLEMENTARY FILE S1.**

**Model construction**

This study employs a three-stage statistical analysis strategy to investigate the effects of 21 trace metal elements and their mixtures in both partners on the early embryological outcomes of couples undergoing IVF (Supplementary Figure S1).

First, considering the complex associations between trace metal elements within and between couples, as well as the potential impact of noise from congeners that were not associated with IVF early embryological outcomes on the joint effects of multiple trace metals, we employed elastic net regression (ENR) to identify the trace metal elements in each partner associated with IVF early embryological outcomes, and subsequently constructed partner-specific models as well as couple-based models based on these findings. Elastic Net Regression (ENR) combines the least absolute shrinkage and selection operator (LASSO, L1 penalty) and ridge (L2 penalty) algorithms, allowing effective feature selection in the presence of highly correlated features (Friedman et al., 2010, Zou and Hastie, 2005). Previous studies have demonstrated that ENR performs well in identifying key chemicals associated with the health impacts of various pollutants (Liu et al., 2022, Yu et al., 2022). In ENR analysis, the penalty strength is adjusted by the parameter α, which balances the gap between LASSO regression (α = 1) and Ridge regression (α = 0), while the overall penalty intensity is controlled by the tuning parameter γ. In this study, the concentrations of trace metals were scaled prior to the ENR analysis, which involved mean centering followed by division by the standard deviation. Finally, the optimal tuning parameters and penalty strengths, which minimize the Root Mean Squared Error (RMSE), were determined through 10-fold cross-validation.

Second, in order to analyze the impact of population exposure patterns on the outcome, we used K-medoids clustering to classify couples and both partners into different exposure groups. In brief, K-medoids clustering is an unsupervised learning algorithm that selects actual samples from the dataset as cluster centers, reducing sensitivity to noise and outliers. Compared to the K-means algorithm, K-medoids uses actual data points as cluster centers, making it more robust to outliers and noisy data points(Yao et al., 2021). Specifically, K-medoids clustering was used to divide both partners (male and female) into two subgroups (high exposure and low exposure) based on the concentrations of 21 trace metal elements in plasma. In the couple-based analysis, based on the exposure levels of both partners, couples were further divided into three subgroups (high exposure, medium exposure, and low exposure). The criteria for defining couples’ exposure were as follows:

1. If both partners were classified as high exposure, the couple was categorized as high exposure.

2. If only one partner was classified as high exposure, the couple was categorized as medium exposure.

3. If both partners were classified as low exposure, the couple was categorized as low exposure.

Additionally, quantile-based g-computation (QGC) and group weighted quantile sum regression (groupWQS) were used to assess the impact of the trace metal mixtures identified by ENR on early IVF embryological outcomes. QGC was employed to evaluate the mixture effects of the exposures. QGC utilizes quantile regression techniques (such as quantile regression or quantile instrumental variable regression) to model the relationship between exposures and outcomes, and then evaluates the causal impact of the exposures on the outcomes by intervening on the exposure factors(Keil et al., 2020, Smarr et al., 2021). Specifically, before conducting the analysis, the trace metals selected by ENR were divided into quartiles. We performed 100 bootstrap iterations to obtain estimates of the mixture effects and the weight estimates for the included trace metals, thereby indirectly assessing the relative importance of each individual trace metal. Moreover, to further evaluate the impact of the grouped trace metal mixtures identified by ENR on the outcomes, groupWQS was also applied. In brief, groupWQS is used to assess the combined effect of multiple exposure groups on a given outcome by analyzing the weights and effect sizes of each exposure group, thus evaluating the contribution of each group to the outcome variable(Wheeler et al., 2021). Therefore, based on the direction of the associations between different trace metal elements and the outcome from the previous QGC results, we further classify the trace metal mixtures identified by ENR into positive and negative effect groups. The weight distribution and effect sizes for each group are then estimated through 100 bootstrap iterations.

Finally, a conventional generalized linear mixed model (GLMM) was applied to construct couple-based models and partner-specific models to analyze the association between individual trace metal elements identified by ENR and early IVF embryological outcomes.

**References**

Friedman J, Hastie T, Tibshirani R. Regularization Paths for Generalized Linear Models via Coordinate Descent. *Journal of statistical software* 2010;33: 1-22.

Keil AP, Buckley JP, O'Brien KM, Ferguson KK, Zhao S, White AJ. A Quantile-Based g-Computation Approach to Addressing the Effects of Exposure Mixtures. *Environmental health perspectives* 2020;128: 47004.

Liu X, Luo K, Zhang J, Yu H, Chen D. Exposure of Preconception Couples to Legacy and Emerging Per- and Polyfluoroalkyl Substances: Variations Within and Between Couples. *Environmental science & technology* 2022;56: 6172-6181.

Smarr MM, Mirzaei Salehabadi S, Boyd Barr D, Buck Louis GM, Sundaram R. A multi-pollutant assessment of preconception persistent endocrine disrupting chemicals and incident pregnancy loss. *Environment international* 2021;157: 106788.

Wheeler DC, Rustom S, Carli M, Whitehead TP, Ward MH, Metayer C. Assessment of Grouped Weighted Quantile Sum Regression for Modeling Chemical Mixtures and Cancer Risk. *International journal of environmental research and public health* 2021;18.

Yao X, Steven Xu X, Yang Y, Zhu Z, Zhu Z, Tao F, Yuan M. Stratification of population in NHANES 2009–2014 based on exposure pattern of lead, cadmium, mercury, and arsenic and their association with cardiovascular, renal and respiratory outcomes. *Environment international* 2021;149: 106410.

Yu L, Liu W, Wang X, Ye Z, Tan Q, Qiu W, Nie X, Li M, Wang B, Chen W. A review of practical statistical methods used in epidemiological studies to estimate the health effects of multi-pollutant mixture. *Environmental pollution (Barking, Essex : 1987)* 2022;306: 119356.

Zou H, Hastie T. Regularization and Variable Selection Via the Elastic Net. *Journal of the Royal Statistical Society Series B: Statistical Methodology* 2005;67: 301-320.


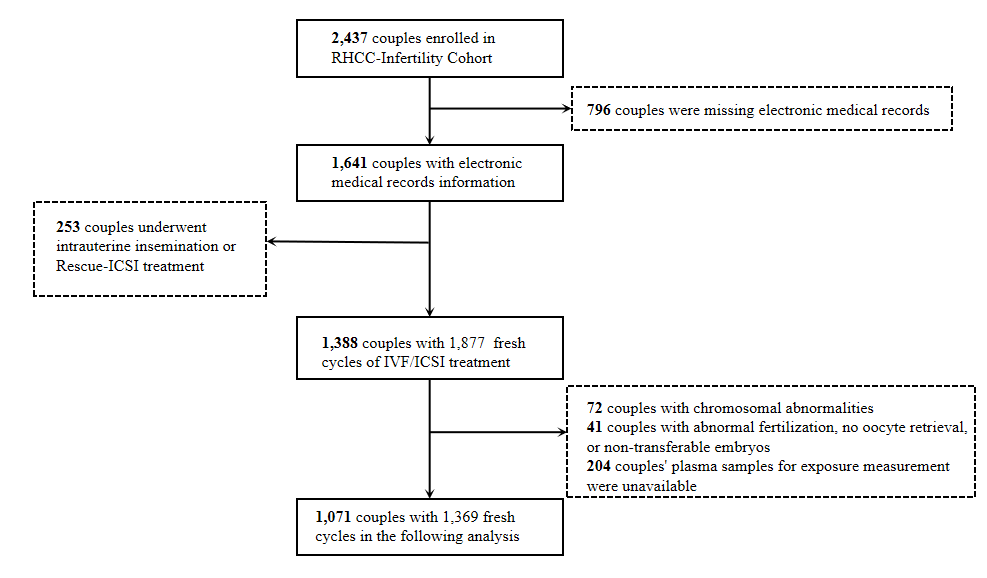


**Supplementary Figure S1.** **The flowchart of study participants included in the present study.** RHCC, the Reproductive Health of Childbearing Couples-Anhui Cohort; Notes: IVF, in vitro fertilization; ICSI, intracytoplasmic sperm injection

**
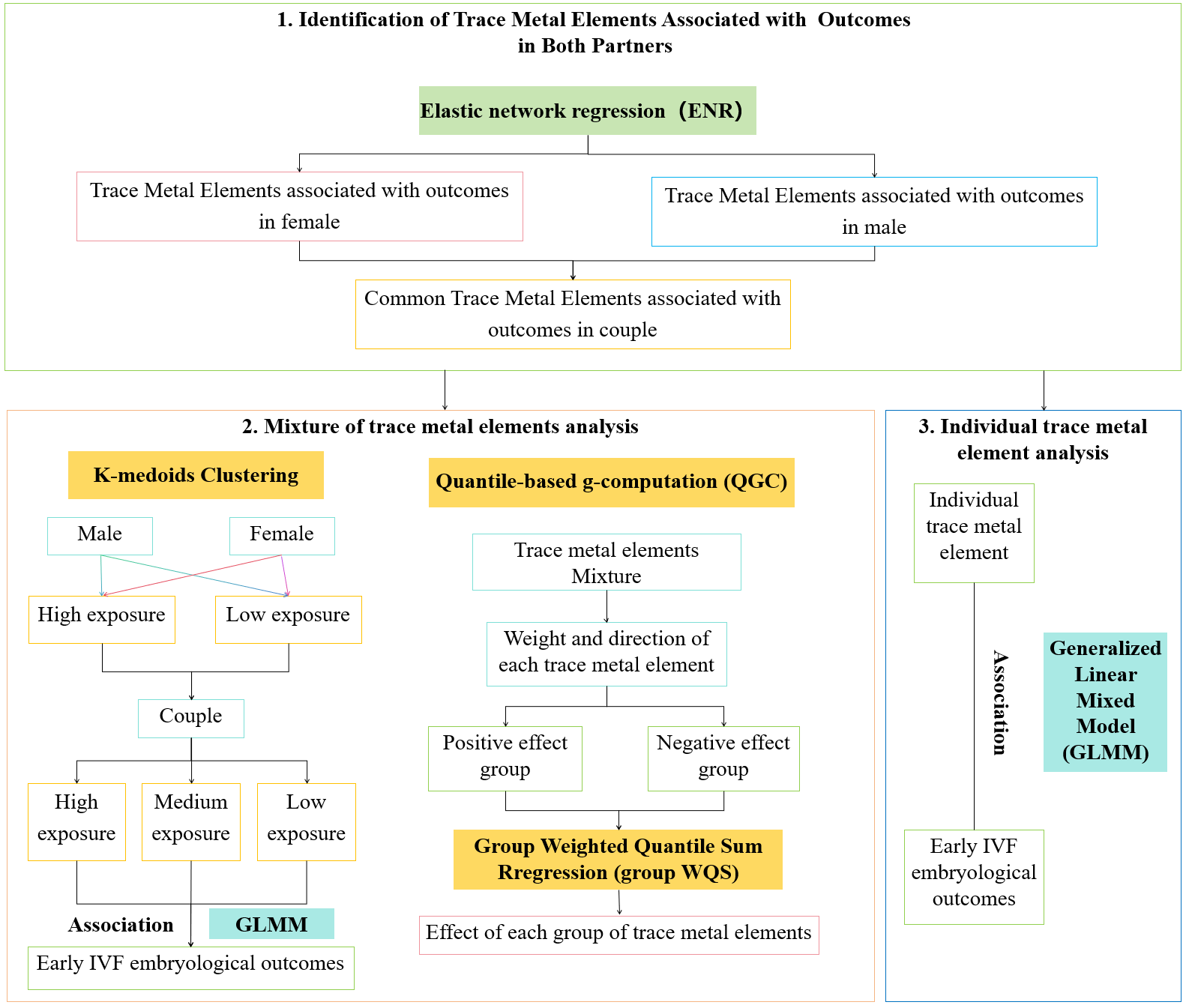
**

**Supplementary Figure S2. Detailed information on statistical modelling.** IVF, *in vitro* fertilization; ENR, elastic network regression; QGC, Quantile based g-computation; groupWQS, group weighted quantile sum regression; GLMM, generalized linear mixed model.


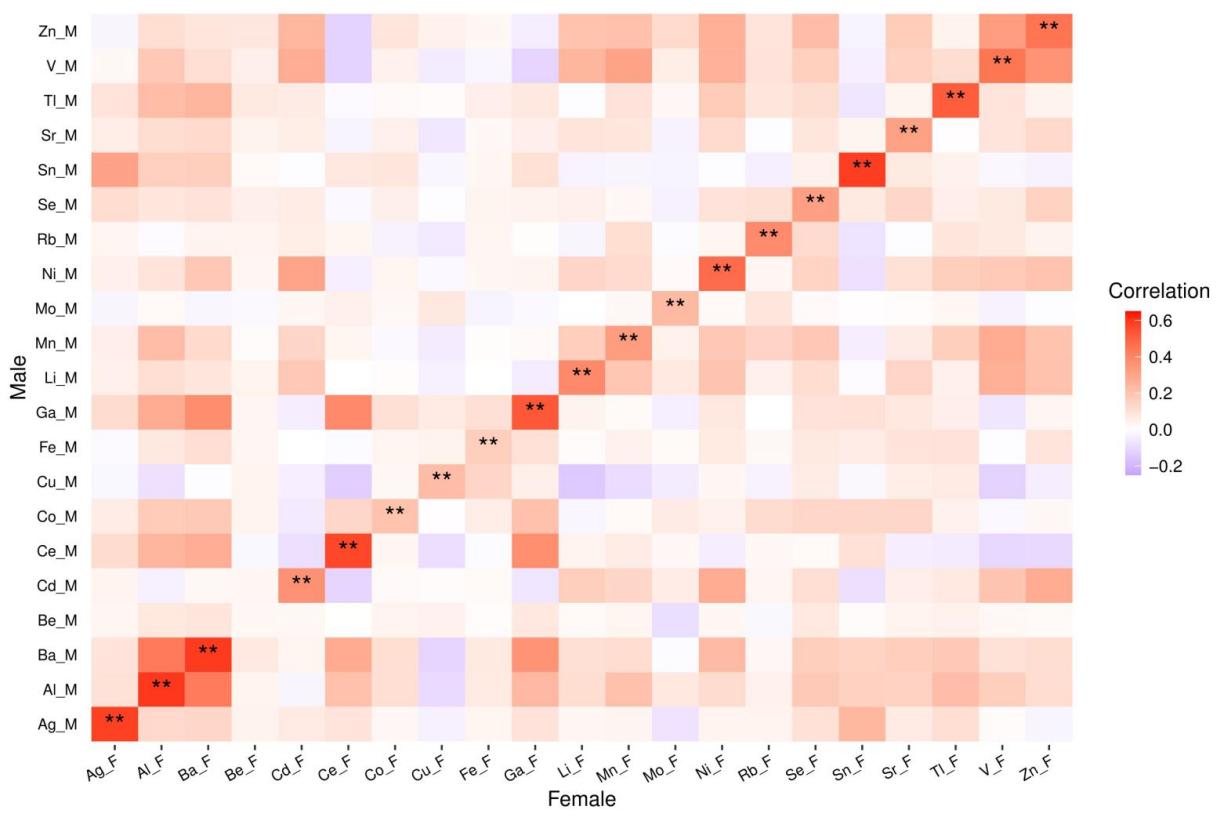


**Supplementary Figure S3** **Spearman correlation matrix of trace metal elements within each couple.** M and F represented the trace metal elements in males and females, respectively.


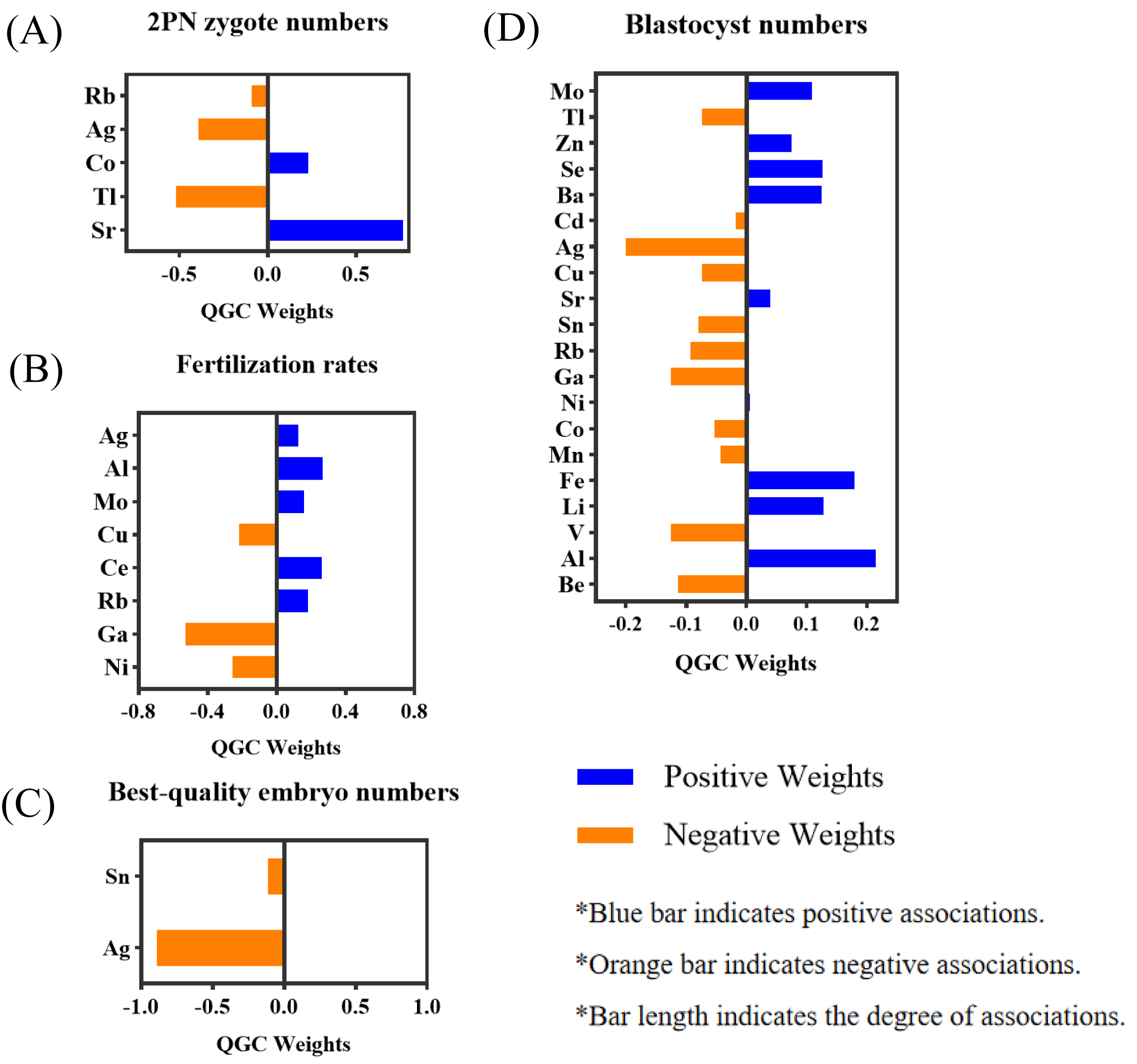


**Supplementary Figure S4. Associations between ENR selected trace metal element mixtures and early embryological outcomes of IVF in males (QGC).** Quantile based g-computation (QGC) method was used for the mixture analysis. Estimates were adjusted for male age, BMI, smoking and drinking status, sperm concentration and season of sampling. For (A) to (D), represents the weights and directions of the associations between trace metal elements and the number of 2PN zygotes, fertilization rates, the number of best-quality embryos, and blastocyst numbers in the QGC analysis. ENR, elastic network regression; IVF, *in vitro* fertilization; CI, confidence interval; RR, relative risk; 2PN, two-pronuclear


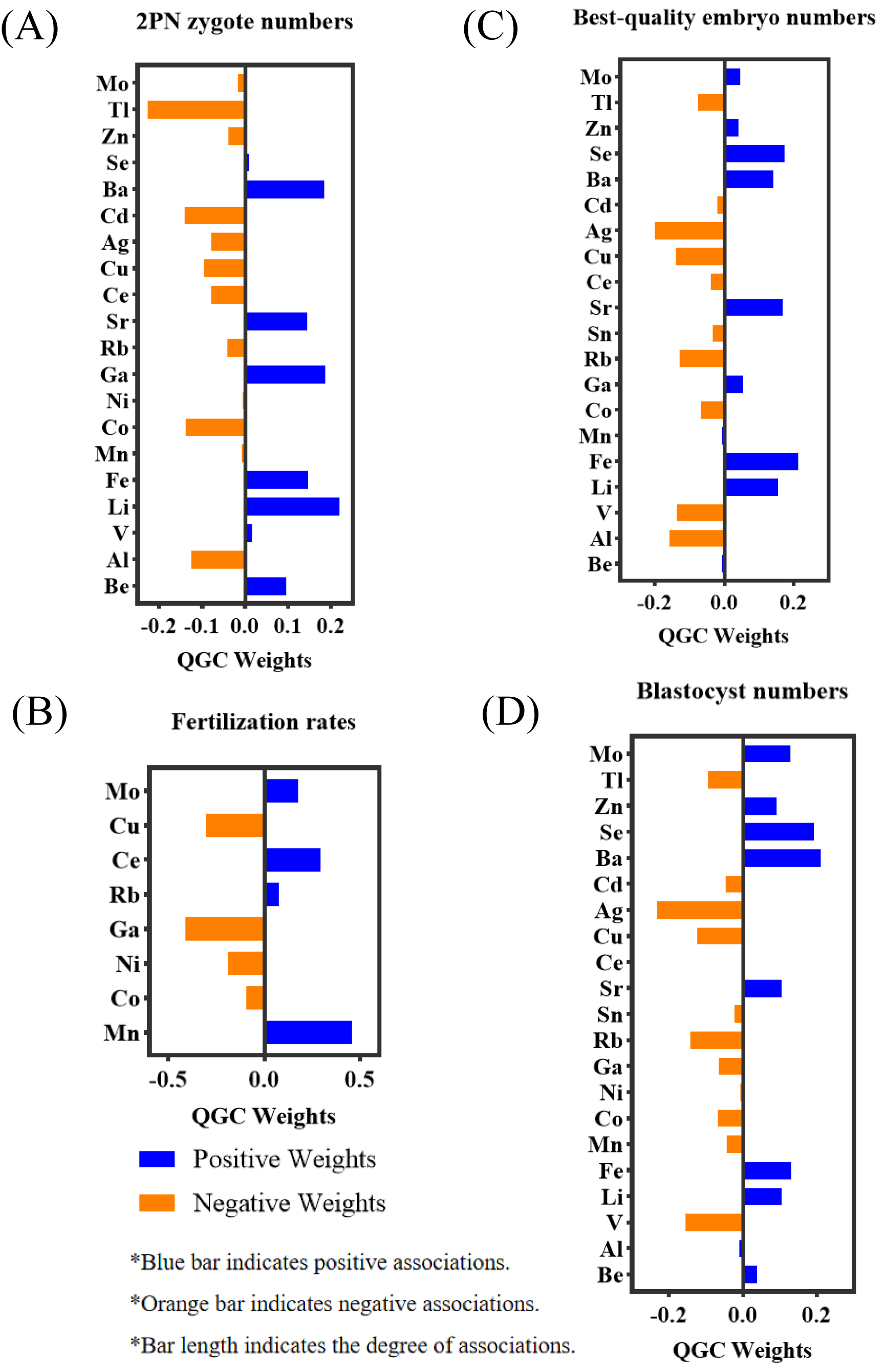


**Supplementary Figure S5.** **Associations between ENR selected trace metal element mixtures and early embryological outcomes of IVF in females (QGC).** Quantile based g-computation (QGC) method was used for the mixture analysis. Estimates were adjusted for female age, BMI, smoking and drinking status, parity, FSH levels, ovulation stimulation protocols and season of sampling. For (A) to (D), represents the weights and directions of the associations between trace metal elements and the number of 2PN zygotes, fertilization rates, the number of best-quality embryos, and blastocyst numbers in the QGC analysis. ENR, elastic network regression; IVF, *in vitro* fertilization; CI, confidence interval; RR, relative risk; 2PN, two-pronuclear


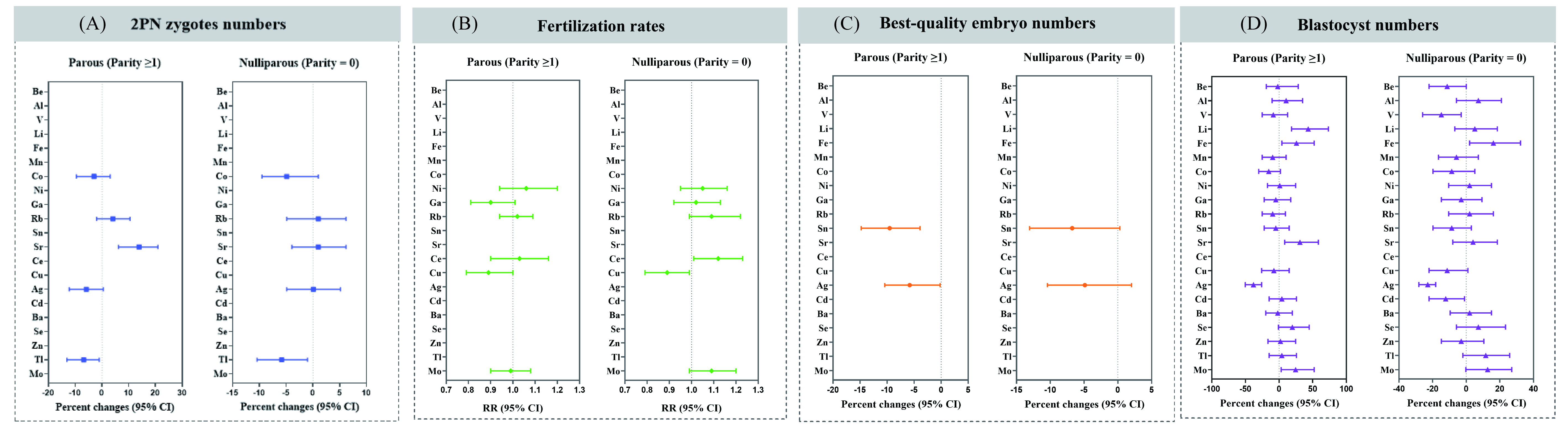


**Supplementary Figure S6. Associations between ENR selected individual trace metal elements with early IVF embryological outcomes was stratified by parity.** The analyses were conducted using generalized linear mixed models with random intercepts. Estimates were adjusted for age, BMI, smoking and drinking status from each partner, parity, sperm concentration, FSH levels, the age difference between couples, ovulation stimulation protocols and season of sampling. For (A), (C), and (D), a poisson distribution and log link function were applied, with data presented as percent changes (95% CI). For (B), a binomial distribution and log link function were utilized, with data presented as RR (95% CI). Notes: ENR, elastic network regression; IVF, *in vitro* fertilization; CI, confidence interval; RR, relative risk; 2PN, two-pronuclear


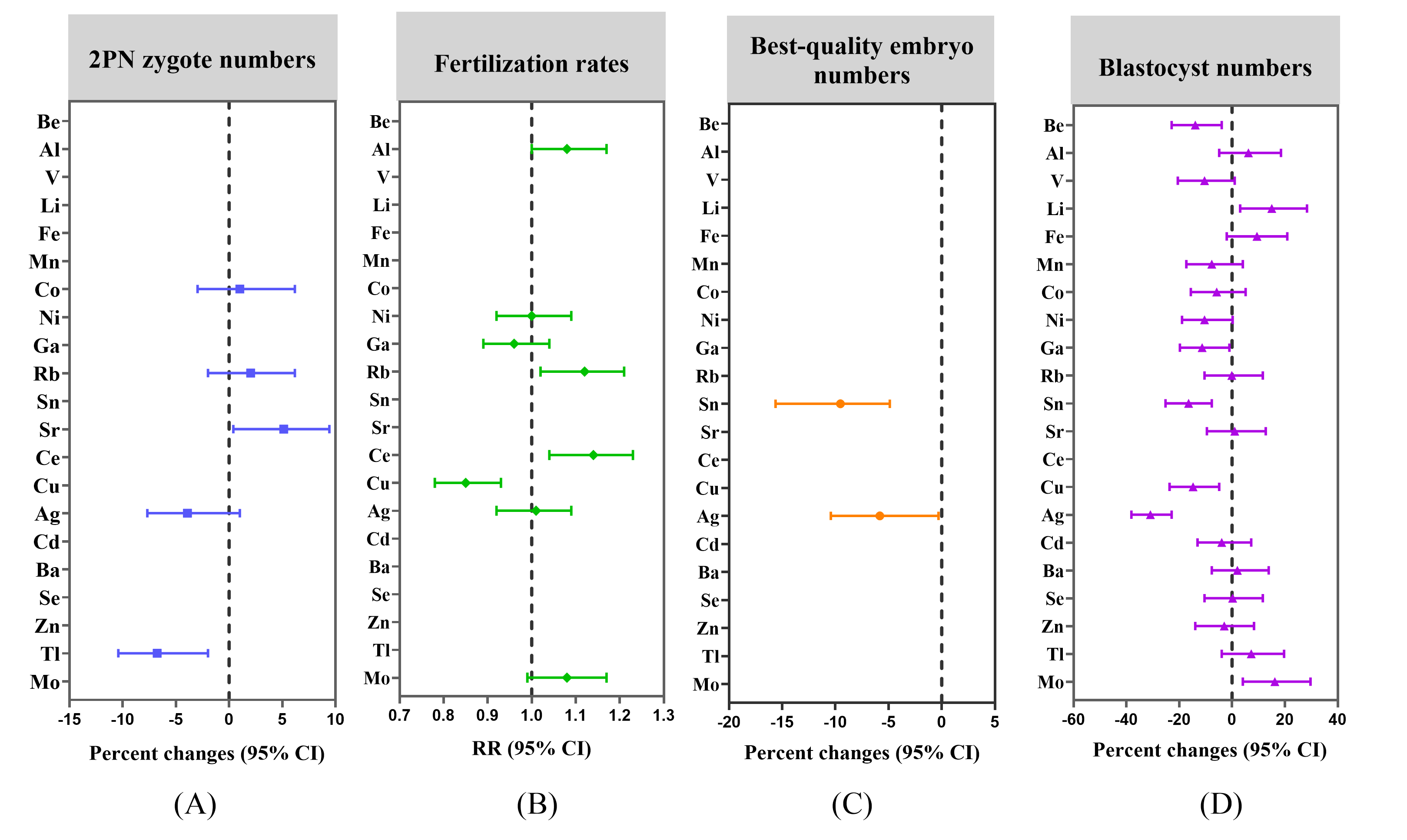


**Supplementary Figure S7. Associations between ENR selected individual trace metal element from with early IVF embryological outcomes in males.** The analyses were conducted using generalized linear mixed models with random intercepts. Estimates were adjusted for male age, BMI, smoking and drinking status, sperm concentration and season of sampling. For (A), (C), and (D), a poisson distribution and log link function were applied, with data presented as percent changes (95% CI). For (B), a binomial distribution and log link function were utilized, with data presented as RR (95% CI). ENR, elastic network regression; IVF, *in vitro* fertilization; BMI, body mass index; CI, confidence interval; RR, relative risk; 2PN, two-pronuclear


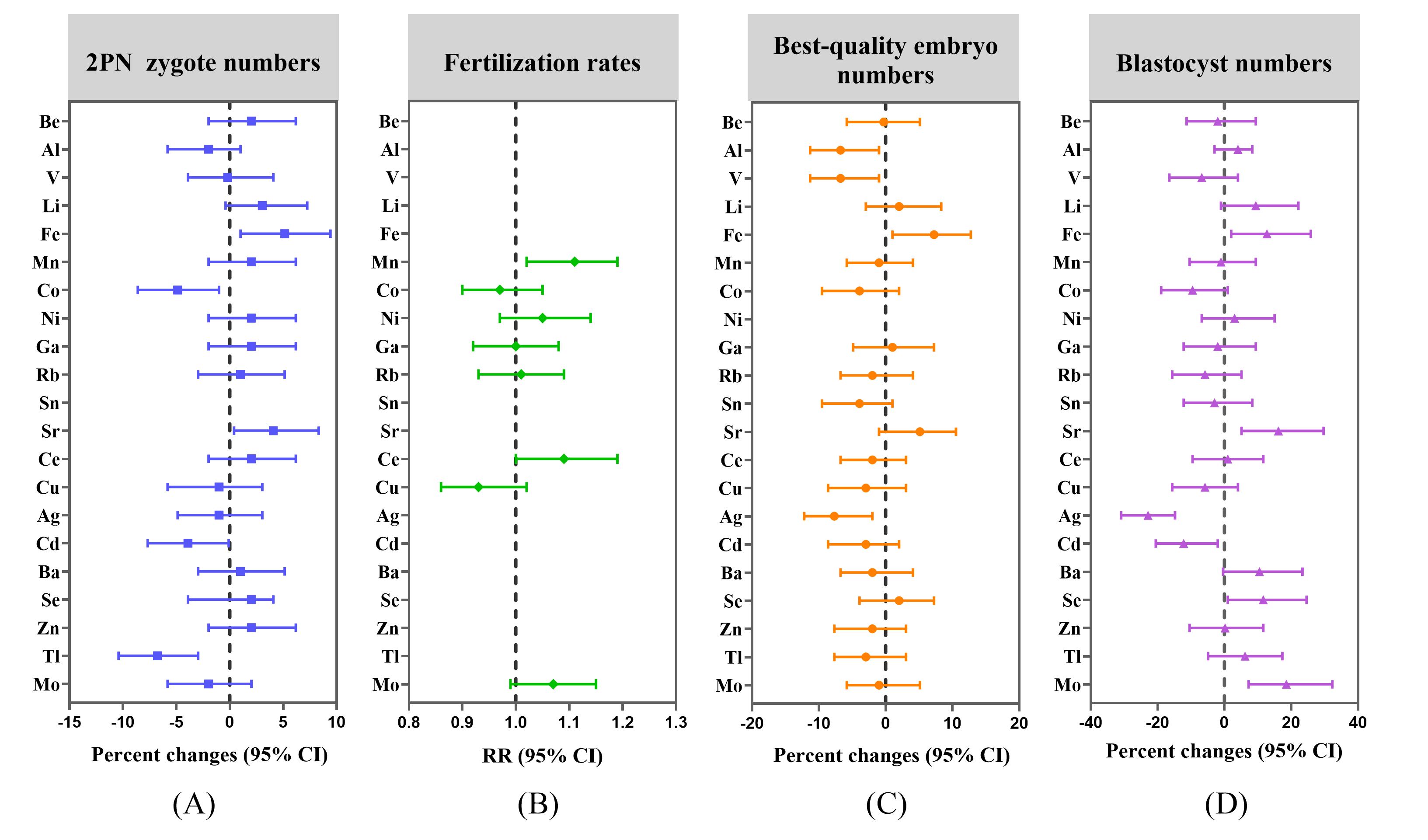


**Supplementary Figure S8. Associations between ENR selected individual trace metal element from with early IVF embryological outcomes in females.** The analyses were conducted using generalized linear mixed models with random intercepts. Estimates were adjusted for female age, BMI, smoking and drinking status, parity, FSH levels, ovulation stimulation protocols and season of sampling. For (A), (C), and (D), a poisson distribution and log link function were applied, with data presented as percent changes (95% CI). For (B), a binomial distribution and log link function were utilized, with data presented as RR (95% CI). ENR, elastic network regression; IVF, *in vitro* fertilization; BMI, body mass index; CI, confidence interval; RR, relative risk; 2PN, two-pronuclear

**Supplementary Table S1.** Detection and distribution of plasma trace metal element concentrations in couples (N=1,071).

| Element | LOD (ng/mL) | N (%) > LOD | Median (Q1, Q3) |
| --- | --- | --- | --- |
| **Male** |  |  |  |
| Be | 0.04702 | 582 (54.3) | 0.13(0.03,0.21) |
| Al | 0.68935 | 1,071(100.0) | 67.70(47.76,91.53) |
| V | 0.00003 | 1,071(100.0) | 0.28(0.20,0.40) |
| Li | 0.02264 | 1,068 (99.7) | 3.49(2.07,6.84) |
| Fe | 0.74444 | 1,071(100.0) | 4339.15(3520.61,5256.60) |
| Mn | 0.06224 | 1,071(100.0) | 1.05(0.86,1.34) |
| Co | 0.00230 | 1,071(100.0) | 0.15(0.13,0.17) |
| Ni | 0.04836 | 1,071(100.0) | 1.38(1.10,1.93) |
| Ga | 0.00469 | 1,071(100.0) | 0.52(0.40,0.68) |
| Rb | 0.00191 | 1,071(100.0) | 228.30(200.83,256.42) |
| Sn | 0.66401 | 1,062 (99.2) | 4.02(2.63,6.26) |
| Sr | 0.12808 | 1,071(100.0) | 36.66(31.02,43.76) |
| Ce | 0.07242 | 1,071(100.0) | 150.01(122.83,195.87) |
| Cu | 0.31070 | 1,071(100.0) | 3273.22(2884.43,3691.57) |
| Ag | 0.00429 | 1,071(100.0) | 0.10(0.08,0.17) |
| Cd | 0.00163 | 1,070 (99.9) | 0.05(0.03,0.10) |
| Ba | 0.62839 | 1,071(100.0) | 64.00(50.25,76.88) |
| Se | 0.02477 | 1,071(100.0) | 93.67(83.16,104.82) |
| Zn | 3.46262 | 1,071(100.0) | 4205.55(3298.26,6341.19) |
| Tl | 0.00032 | 1,071(100.0) | 0.13(0.10,0.18) |
| Mo | 0.43904 | 1,071(100.0) | 26.58(21.02,33.15) |
| **Female** |  |  |  |
| Be | 0.04702 | 565 (52.8) | 0.13(0.03,0.21) |
| Al | 0.68935 | 1,071(100.0) | 61.92(43.69,89.81) |
| V | 0.00003 | 1,071(100.0) | 0.25(0.18,0.37) |
| Li | 0.02264 | 1,063 (99.3) | 3.04(1.84,5.84) |
| Fe | 0.74444 | 1,071(100.0) | 3367.83(2584.93,4256.60) |
| Mn | 0.06224 | 1,071(100.0) | 0.98(0.78,1.30) |
| Co | 0.00230 | 1,071(100.0) | 0.22(0.16,0.33) |
| Ni | 0.04836 | 1,071(100.0) | 1.36(1.06,1.85) |
| Ga | 0.00469 | 1,071(100.0) | 0.48(0.38,0.62) |
| Rb | 0.00191 | 1,071(100.0) | 200.56(177.05,224.25) |
| Sn | 0.66401 | 1,061(99.1) | 3.73(2.50,5.75) |
| Sr | 0.12808 | 1,071(100.0) | 36.22(30.15,43.77) |
| Ce | 0.07242 | 1,071(100.0) | 137.80(113.86,176.84) |
| Cu | 0.31070 | 1,071(100.0) | 3771.78(3284.25,4333.18) |
| Ag | 0.00429 | 1,071(100.0) | 0.11(0.08,0.17) |
| Cd | 0.00163 | 1,069 (99.8) | 0.05(0.03,0.08) |
| Ba | 0.62839 | 1,071(100.0) | 58.78(46.18,71.56) |
| Se | 0.02477 | 1,071(100.0) | 88.95(78.74,100.26) |
| Zn | 3.46262 | 1,071(100.0) | 3930.91(3098.58,5775.18) |
| Tl | 0.00032 | 1,071(100.0) | 0.13(0.10,0.17) |
| Mo | 0.43904 | 1,071(100.0) | 25.39(21.13,30.21) |

Only trace metal elements detected in >50 of each partner were included in the final association analyses. LOD: limits of detection; N (%) > LOD: detection rate, referring to the proportion of individuals with plasma trace metal element concentrations above the LOD; Q1 and Q3 refer to the 1st and 3rd quartile.

**Supplementary Table S2.** Associations between trace metal elements and early embryological outcomes of IVF in couple-based elastic network regression (ENR) analyses.

| Trace metal elements | Early embryological outcomes of IVF | | | |
| --- | --- | --- | --- | --- |
|  | 2PN zygote numbers (β) | Fertilization rates (β) | Best-quality embryo numbers (β) | Blastocyst numbers (β) |
| **Male** |  |  |  |  |
| Be | NA | NA | NA | -0.070 |
| Al | NA | 0.009 | NA | 0.266 |
| V | NA | NA | NA | -0.318 |
| Li | NA | NA | NA | 0.110 |
| Fe | NA | NA | NA | 0.326 |
| Mn | NA | NA | NA | -0.031 |
| Co | 0.023 | NA | NA | 0.066 |
| Ni | NA | -0.012 | NA | -0.155 |
| Ga | NA | -0.067 | NA | -0.455 |
| Rb | 0.066 | 0.068 | NA | -0.027 |
| Sn | NA | NA | -0.034 | -0.110 |
| Sr | 0.038 | NA | NA | 0.105 |
| Ce | NA | 0.064 | NA | NA |
| Cu | NA | -0.066 | NA | -0.322 |
| Ag | -0.022 | 0.006 | 0.056 | -0.448 |
| Cd | NA | NA | NA | 0.019 |
| Ba | NA | NA | NA | 0.328 |
| Se | NA | NA | NA | 0.319 |
| Zn | NA | NA | NA | 0.057 |
| Tl | -0.063 | NA | NA | -0.003 |
| Mo | NA | 0.036 | NA | 0.190 |
| **Female** |  |  |  |  |
| Be | 0.027 | NA | 0.010 | 0.018 |
| Al | -0.095 | NA | -0.165 | -0.055 |
| V | -0.095 | NA | -0.277 | -0.346 |
| Li | 0.003 | NA | 0.034 | 0.084 |
| Fe | 0.061 | NA | 0.181 | 0.176 |
| Mn | 0.085 | 0.041 | 0.113 | 0.074 |
| Co | -0.143 | -0.005 | -0.109 | -0.255 |
| Ni | 0.036 | -0.002 | NA | 0.071 |
| Ga | -0.010 | -0.056 | 0.105 | -0.419 |
| Rb | 0.181 | 0.020 | -0.056 | -0.044 |
| Sn | NA | NA | -0.017 | -0.037 |
| Sr | 0.174 | NA | 0.294 | 0.454 |
| Ce | -0.009 | 0.073 | -0.114 | 0.222 |
| Cu | -0.094 | -0.053 | -0.240 | -0.402 |
| Ag | -0.044 | NA | -0.157 | -0.342 |
| Cd | -0.071 | NA | -0.006 | -0.069 |
| Ba | 0.176 | NA | 0.145 | 0.376 |
| Se | -0.127 | NA | 0.022 | 0.443 |
| Zn | 0.146 | NA | 0.165 | 0.212 |
| Tl | -0.151 | NA | -0.028 | -0.001 |
| Mo | -0.072 | 0.027 | -0.034 | 0.204 |

IVF, *in vitro* fertilization; ENR, elastic network regression; 2PN, two-pronuclear; “NA” denotes trace elements that were found to have no significant association with the outcomes in the ENR analysis.

**Supplementary Table S3.** Associations between ENR selected individual trace metal element and 2PN zygote numbers in nulliparous and parous couples.

| Trace metal elements  (Couple) | 2PN zygote numbers^a^  percent changes (95% CI) | | |
| --- | --- | --- | --- |
|  | Parous (Parity ≥1) | Nulliparous (Parity = 0) | *P* for interaction |
| Co | -2.96(-9.52,3.05) | -4.88(-9.52,1.01) | 0.69 |
| Rb | 4.08(-1.98,10.52) | 1.01(-4.88,6.18) | 0.16 |
| Sr | 13.88(6.18,20.92) | 1.01(-3.92,6.18) | 0.07 |
| Ag | -5.82(-12.19,0.50) | 0.10(-4.88,5.13) | 0.77 |
| Tl | -6.76(-13.06,-1.00) | -5.82(-10.42,-1.00) | 0.01 |

^a^Data are presented as percent changes (95% CI). ENR, elastic network regression; CI, confidence interval; 2PN, two-pronuclear;

**Supplementary Table S4.** Associations between ENR selected individual trace metal element and fertilization rates in nulliparous and parous couples.

| Trace metal elements  (Couple) | Fertilization rates^a^  RR (95% CI) | | |
| --- | --- | --- | --- |
|  | Parous (Parity ≥1) | Nulliparous (Parity = 0) | *P* for interaction |
| Ni | 1.06(0.94,1.20) | 1.05(0.95,1.16) | 0.66 |
| Ga | 0.90(0.81,1.01) | 1.02(0.92,1.13) | 0.18 |
| Rb | 1.02(0.94,1.09) | 1.09(0.99,1.22) | 0.79 |
| Ce | 1.03(0.90,1.16) | 1.12(1.01,1.23) | 0.19 |
| Cu | 0.89(0.79,1.00) | 0.89(0.79,0.99) | 0.97 |
| Mo | 0.99(0.90,1.08) | 1.09(0.99,1.20) | 0.19 |

^a^Data are presented as RR (95% CI). ENR, elastic network regression; CI, confidence interval; RR, relative risk

**Supplementary Table S5.** Associations between ENR selected individual trace metal element and best-quality embryo numbers in nulliparous and parous couples.

| Trace metal elements  (Couple) | Best-quality embryo numbers^a^  percent changes (95% CI) | | |
| --- | --- | --- | --- |
|  | Parous (Parity ≥1) | Nulliparous (Parity = 0) | *P* for interaction |
| Sn | -9.52(-14.79,-3.92) | -6.76(-13.06,0.30) | 0.99 |
| Ag | -5.82(-10.42,-0.20) | -4.88(-10.42,2.02) | 0.36 |

^a^Data are presented as percent changes (95% CI). ENR, elastic network regression; CI, confidence interval

**Supplementary Table S6.** Associations between ENR selected individual trace metal element and blastocyst numbers in nulliparous and parous couples.

| Trace metal elements  (Couple) | Blastocyst numbers^a^  percent changes (95% CI) | | |
| --- | --- | --- | --- |
|  | Parous (Parity ≥1) | Nulliparous (Parity = 0) | *P* for interaction |
| Be | -1.98(-18.94,28.53) | -11.31(-22.12,0.03) | 0.78 |
| Al | 10.52(-10.42,34.99) | 7.25(-5.82,20.92) | 0.61 |
| V | -8.61(-25.17,12.75) | -14.79(-25.92,-2.96) | 0.17 |
| Li | 43.33(18.53,73.33) | 5.13(-6.76,18.53) | 0.41 |
| Fe | 25.86(4.08,52.20) | 16.18(2.02,32.31) | 0.84 |
| Mn | -9.52(-25.17,10.52) | -5.82(-16.47,7.25) | 0.35 |
| Co | -15.36(-30.23,2.02) | -8.61(-19.75,5.13) | 0.34 |
| Ni | 1.01(-17.30,24.61) | 2.02(-10.42,15.03) | 0.55 |
| Ga | -4.88(-22.12,17.35) | -2.96(-14.79,9.42) | 0.55 |
| Rb | -9.52(-25.17,9.42) | 2.02(-10.42,16.18) | 0.71 |
| Sn | -4.88(-22.12,15.03) | -8.61(-19.75,3.05) | 0.76 |
| Sr | 31.00(8.33,58.41) | 4.08(-7.96,18.53) | 0.31 |
| Cu | -7.69(-25.92,15.03) | -11.31(-22.12,1.01) | 0.47 |
| Ag | -38.12(-50.34,-25.92) | -28.11(-22.89,-18.13) | 0.39 |
| Cd | 4.08(-14.79,25.86) | -12.19(-22.12,-1.00) | 0.26 |
| Ba | -1.98(-19.75,19.72) | 2.02(-9.52,15.03) | 0.51 |
| Se | 19.72(-1.00,44.77) | 7.25(-5.82,23.37) | 0.55 |
| Zn | 2.02(-16.47,24.61) | -2.96(-14.79,10.52) | 0.62 |
| Tl | 4.08(-14.79,25.86) | 11.63(-1.98,25.86) | 0.23 |
| Mo | 24.61(3.05,52.20) | 12.75(-0.20,27.12) | 0.60 |

^a^Data are presented as percent changes (95% CI). ENR, elastic network regression; CI, confidence interval; RR, relative risk

Supplementary Table S7. Associations between ENR selected individual trace metal element with early IVF embryological outcomes in males.

| Early embryological outcomes of IVF | Element | Continuous^c^ | Tertiles^d^ | | |
| --- | --- | --- | --- | --- | --- |
|  |  |  | T1 | T2 | T3 |
| 2PN zygote numbers^a^ | Co | 1.01(-2.69,6.18) | Reference | 3.05(-7.69,13.88) | 2.20(-8.61,13.88) |
|  | Rb | 2.02(-1.98,6.18) | Reference | -5.82(-15.63,4.08) | 1.01(-9.52,11.63) |
|  | Sr | 5.13(0.40,9.42)* | Reference | -4.88(-14.79,5.13) | 10.52(-1.00,22.14) |
|  | Ag | -3.92(-7.69,1.01) | Reference | -2.96(-10.42,10.52) | -5.82(-15.63,5.13) |
|  | Tl | -6.76(-10.42,-1.98)* | Reference | -6.76(-15.63,4.08) | -12.19(-20.55,-1.98)* |
| Fertilization rates^b^ | Al | 1.08(1.00,1.17) | Reference | 1.13(0.92,1.18) | 1.27(1.04,1.55)* |
|  | Ni | 1.00(0.92,1.09) | Reference | 0.92(0.76,1.12) | 0.92(0.75,1.14) |
|  | Ga | 0.96(0.89,1.04) | Reference | 0.68(0.56,0.84)* | 0.76(0.63,0.93)* |
|  | Rb | 1.12(1.02,1.21)* | Reference | 1.13(0.92,1.36) | 1.21(0.99,1.48) |
|  | Ce | 1.14(1.04,1.23)* | Reference | 1.09(0.90,1.34) | 1.30(1.07,1.57) |
|  | Cu | 0.85(0.78,0.93)* | Reference | 0.84(0.68,1.02) | 0.66(0.54,0.81)* |
|  | Ag | 1.01(0.92,1.09) | Reference | 1.25(1.02,1.52) | 1.12(0.91,1.36) |
|  | Mo | 1.08(0.99,1.17) | Reference | 1.08(0.89,1.20) | 1.12(0.90,1.36) |
| Best-quality embryo numbers^a^ | Sn | -9.52(-15.63,-4.88)* | Reference | -14.79(-25.92,-1.98)* | -13.06(-24.42,-0.20)* |
|  | Ag | -5.82(-10.42,-0.30)* | Reference | -9.52(-21.34,3.05) | -24.42(-34.30,-13.06)* |
| Blastocyst numbers^a^ | Be | -13.93(-22.89, -3.92)* | Reference | -18.94(-39.35,8.33) | -25.92(-42.31,-5.82)* |
|  | Al | 6.18 (-4.88, 18.53) | Reference | 11.63(-14.79,46.23) | 27.12(-1.98,66.53) |
|  | V | -10.42 (-20.55, 1.01) | Reference | 13.88(-13.06,49.18) | -4.88(-28.11,25.86) |
|  | Li | 15.03 (3.05, 28.40)* | Reference | -1.98(-24.42,28.40) | 22.14(-6.76,60.00) |
|  | Fe | 9.42 (-1.98, 20.92) | Reference | 9.42(-15.63,41.91) | 12.75(-13.93,47.70) |
|  | Mn | -7.69 (-17.30, 4.08) | Reference | 3.05(-21.34,34.99) | 10.52(-15.63,44.77) |
|  | Co | -5.82 (-15.63, 5.13) | Reference | 9.42(-15.63,43.33) | -14.79(-35.60,12.75) |
|  | Ni | -10.42(-18.94,0.20) | Reference | 25.86(-2.96,28.40) | -15.63(-36.24,10.52) |
|  | Ga | -11.31(-19.75,-1.00)* | Reference | -36.24(-50.84,-16.47)* | -23.66(-41.14,-1.00)* |
|  | Rb | -0.10(-10.42,11.63) | Reference | -6.76(-28.11,22.14) | -19.75(-38.74,5.13) |
|  | Sn | -16.47(-25.17,-7.69)* | Reference | -4.88(-26.66,23.37) | -39.35(-53.70,-20.55)* |
|  | Sr | 1.01(-9.52,12.75) | Reference | -6.76(-28.82,22.14) | 7.25(-18.13,40.49) |
|  | Cu | -14.79(-23.66,-4.88)* | Reference | -10.42(-30.93,16.18) | -29.53(-46.21,-7.69)* |
|  | Ag | -30.93(-38.12,-22.89)* | Reference | -11.31(-30.93,13.88) | -62.09(-71.06,-50.34)* |
|  | Cd | -3.92(-13.06,7.25) | Reference | 2.02(-22.12,33.64) | -17.30(-37.50,8.33) |
|  | Ba | 2.02(-7.69,13.88) | Reference | 8.33(-17.30,41.91) | -2.96(-25.17,25.86) |
|  | Se | 0.20(-10.42,11.63) | Reference | 36.34(5.13,78.60)* | 5.13(-19.75,37.71) |
|  | Zn | -2.96(-13.93,8.33) | Reference | 0.30(-22.89,31.00) | 23.37(-4.88,61.61) |
|  | Tl | 7.25(-3.92,19.72) | Reference | -0.20(-23.66,29.69) | -16.47(-36.24,9.42) |
|  | Mo | 16.18(4.08,29.69)* | Reference | 20.92(-7.69,60.00) | 46.23(11.63,91.55)* |

^a^Data are presented as percent changes (95% CI). ^b^Data are presented as RR (95% CI). ^c^Elements were modeled as continuous variables in the multivariate generalized linear mixed model. ^d^Elements were modeled as categorical variables (tertiles) in the multivariate generalized linear mixed model. ENR, elastic network regression; IVF, *in vitro* fertilization; CI, confidence interval; RR, relative risk; 2PN, two-pronuclear; * *P*-value <0.05.

**Supplementary Table S8.** Associations between ENR selected individual trace metal element with early IVF embryological outcomes in females.

| Early embryological outcomes of IVF | Element | Continuous^c^ | Tertiles^d^ | | |
| --- | --- | --- | --- | --- | --- |
|  |  |  | T1 | T2 | T3 |
| 2PN zygote numbers^a^ | Be | 2.02(-1.98,6.18) | Reference | -0.30(-10.42,10.52) | -1.00(-7.69,10.52) |
|  | Al | -1.98(-5.82,1.01) | Reference | 1.01(-8.61,11.63) | -5.82(-14.79,3.05) |
|  | V | -0.20(-3.92,4.08) | Reference | -1.98(-11.31,8.33) | -3.92(-13.06,6.18) |
|  | Li | 3.05(-0.40,7.25) | Reference | 2.02(-7.69,12.75) | 11.63(1.01,22.14)* |
|  | Fe | 5.13(1.01,9.42)* | Reference | 10.52(0.00,20.92)* | 7.25(-2.96,18.53) |
|  | Mn | 2.02(-1.98,6.18) | Reference | -3.92(-13.06,6.18) | -1.98(-11.31,8.33) |
|  | Co | -4.88(-8.61,-1.00)* | Reference | -4.88(-13.93,4.08) | -11.31(-19.75,-1.98)* |
|  | Ni | 2.02(-1.98,6.18) | Reference | 1.01(-8.61,10.52) | 2.02(-7.69,12.75) |
|  | Ga | 2.02(-1.98,6.18) | Reference | 2.02(-7.69,12.75) | 7.25(-1.98,19.72) |
|  | Rb | 1.01(-2.96,5.13) | Reference | -9.52(-18.13,0.02) | -1.98(-11.31,7.25) |
|  | Sr | 4.08(0.40,8.33)* | Reference | 0.50(-8.61,10.52) | 7.25(-2.96,18.53) |
|  | Ce | 2.02(-1.98,6.18) | Reference | -1.00(-10.42,9.42) | 9.42(-1.00,20.92) |
|  | Cu | -1.00(-5.82,3.05) | Reference | -0.20(-9.52,10.52) | -5.82(-15.63,4.08) |
|  | Ag | -1.00(-4.88,3.05) | Reference | -5.82(-13.93,4.08) | -1.98(-11.31,8.33) |
|  | Cd | -3.92(-7.69,-0.10)* | Reference | -10.42(-18.13,-1.00)* | -8.61(-17.30,1.01) |
|  | Ba | 1.01(-2.96,5.13) | Reference | 6.18(-3.92,17.35) | 5.13(-4.88,16.18) |
|  | Se | 2.02(-3.92,4.08) | Reference | -6.76(-15.63,2.02) | -1.98(-11.31,7.25) |
|  | Zn | 2.02(-1.98,6.18) | Reference | -1.98(-11.31,8.33) | 0.40(-8.61,10.52) |
|  | Tl | -6.76(-10.42,-2.96)* | Reference | -2.96(-11.31,7.25) | -13.06(-20.55,-3.92)* |
|  | Mo | -1.98(-5.82,2.02) | Reference | -1.00(-10.42,8.33) | -5.82(-14.79,4.08) |
| Fertilization rates^b^ | Mn | 1.11(1.02,1.19)* | Reference | 1.07(0.89,1.31) | 1.27(1.04,1.54)* |
|  | Co | 0.97(0.90,1.05) | Reference | 1.04(0.86,1.27) | 0.95(0.78,1.15) |
|  | Ni | 1.05(0.97,1.14) | Reference | 1.15(0.95,1.39) | 1.11(0.90,1.34) |
|  | Ga | 1.00(0.92,1.08) | Reference | 0.88(0.72,1.06) | 0.97(0.79,1.19) |
|  | Rb | 1.01(0.93,1.09) | Reference | 1.00(0.83,1.22) | 0.98(0.80,1.19) |
|  | Ce | 1.09(1.00,1.19)* | Reference | 1.08(0.89,1.31) | 1.23(1.02,1.51)* |
|  | Cu | 0.93(0.86,1.02) | Reference | 0.94(0.77,1.15) | 0.84(0.69,1.04) |
|  | Mo | 1.07(0.99,1.15) | Reference | 1.03(0.85,1.25) | 1.16(0.95,1.42) |
| Best-quality embryo numbers^a^ | Be | -0.30(-5.82,5.13) | Reference | -2.96(-16.47,12.75) | -1.00(-12.19,11.63) |
|  | Al | -6.76(-11.31,-1.00)* | Reference | -3.92(-15.63,9.42) | -15.63(-25.92,-2.96)* |
|  | V | -6.76(-11.31,-1.00)* | Reference | -10.42(-21.34,3.05) | -15.63(-25.92,-2.96)* |
|  | Li | 2.02(-2.96,8.33) | Reference | -1.98(-14.79,11.63) | 3.05(-9.52,17.35) |
|  | Fe | 7.25(1.01,12.75)* | Reference | 16.18(1.01,32.31)* | 15.03(1.01,32.31)* |
|  | Mn | -1.00(-5.82,4.08) | Reference | -1.98(-14.79,11.63) | -6.76(-18.94,6.18) |
|  | Co | -3.92(-9.52,2.02) | Reference | 2.02(-10.42,16.18) | -7.69(-19.75,6.18) |
|  | Ga | 1.01(-4.88,7.25) | Reference | 4.08(-8.61,19.72) | 7.25(-6.76,23.37) |
|  | Rb | -1.98(-6.76,4.08) | Reference | -12.19(-23.66,0.40) | -7.69(-18.94,6.18) |
|  | Sn | -3.92(-9.52,1.01) | Reference | -19.75(-29.53,-7.69)* | -13.06(-23.66,-0.20)* |
|  | Sr | 5.13(-1.00,10.52) | Reference | 9.42(-4.88,24.61) | 8.33(-5.82,24.61) |
|  | Ce | -1.98(-6.76,3.05) | Reference | -6.76(-18.94,6.18) | 0.40(-12.19,15.03) |
|  | Cu | -2.96(-8.61,3.05) | Reference | 0.30(-12.19,15.03) | -5.82(-18.13,9.42) |
|  | Ag | -7.69(-12.19,-1.98)* | Reference | -12.19(-23.66,0.02) | -14.79(-25.92,-2.96)* |
|  | Cd | -2.96(-8.61,2.02) | Reference | 3.05(-10.42,17.35) | -6.76(-18.94,6.18) |
|  | Ba | -1.98(-6.76,4.08) | Reference | 3.05(-10.42,17.35) | -1.98(-13.93,12.75) |
|  | Se | 2.02(-3.92,7.25) | Reference | -7.69(-18.94,5.13) | -0.40(-13.06,13.88) |
|  | Zn | -1.98(-7.69,3.05) | Reference | 9.42(-4.88,25.86) | 4.08(-9.52,18.53) |
|  | Tl | -2.96(-7.69,3.05) | Reference | -8.61(-19.75,5.13) | -13.93(-24.42,-1.98)* |
|  | Mo | -1.00(-5.82,5.13) | Reference | 11.63(-22.89,27.12) | -0.10(-13.06,15.03) |
| Blastocyst numbers^a^ | Be | -1.98(-11.31,9.42) | Reference | -32.97(-49.84,-11.31)* | 5.13(-16.47,33.64) |
|  | Al | 7.25(-2.96,8.33) | Reference | 22.14(-4.88,58.41) | 5.13(-18.94,36.34) |
|  | V | -6.76(-16.47,4.08) | Reference | -13.93(-33.63,11.63) | -18.94(-37.50,5.13) |
|  | Li | 9.42(-1.00,22.14) | Reference | 11.63(-13.06,44.77) | 20.92(-6.76,55.27) |
|  | Fe | 12.75(2.02,25.86)* | Reference | 26.69(1.01,66.53)* | 27.12(-1.98,64.87) |
|  | Mn | -1.00(-10.42,9.42) | Reference | -6.76(-28.11,19.72) | 6.18(-17.30,37.71) |
|  | Co | -9.52(-18.94,1.01) | Reference | -2.96(-24.42,24.61) | -23.66(-41.14,-1.00)* |
|  | Ni | 3.05(-6.76,15.03) | Reference | -5.82(-27.39,22.14) | -3.92(-25.92,24.61) |
|  | Ga | -1.98(-12.19,9.42) | Reference | -1.98(-24.42,27.12) | 8.33(-16.47,40.49) |
|  | Rb | -5.82(-15.63,5.13) | Reference | -18.13(-37.50,6.18) | -14.79(-34.95,10.52) |
|  | Sn | -2.96(-12.19,8.33) | Reference | -26.66(-42.88,-5.82)* | -15.63(-34.95,-8.61)* |
|  | Sr | 16.18(5.13,29.69)* | Reference | 2.02(-2.96,61.61) | 33.64(2.02,73.33)* |
|  | Ce | 1.01(-9.52,11.63) | Reference | -10.42(-30.93,16.18) | 32.31(3.05,69.89)* |
|  | Cu | -5.82(-15.63,4.08) | Reference | -3.92(-25.17,24.61) | -14.79(-34.95,11.63) |
|  | Ag | -22.89(-30.93,-14.79)* | Reference | -22.12(-39.35,-0.10)* | -51.81(-62.84,-37.50)* |
|  | Cd | -12.19(-20.55,-1.98)* | Reference | 7.25(-17.30,37.71) | -25.17(-42.31,-2.96)* |
|  | Ba | 10.52(-0.40,23.37) | Reference | 36.34(5.13,76.83)* | 25.86(-2.96,61.61) |
|  | Se | 11.63(1.01,24.61)* | Reference | 18.53(-7.69,53.73) | 37.71(6.18,78.60)* |
|  | Zn | 0.20(-10.42,11.63) | Reference | 23.37(-4.88,60.00) | 33.64(3.05,71.60)* |
|  | Tl | 6.18(-4.88,17.35) | Reference | -22.12(-39.95,-0.10) | -15.63(-34.95,7.25) |
|  | Mo | 18.53(7.25,32.31)* | Reference | 80.40(39.10,131.64)* | 64.87(25.86,113.83)* |

^a^Data are presented as percent changes (95% CI). ^b^Data are presented as RR (95% CI). ^c^Elements were modeled as continuous variables in the multivariate generalized linear mixed model. ^d^Elements were modeled as categorical variables (tertiles) in the multivariate generalized linear mixed model. ENR, elastic network regression; IVF, *in vitro* fertilization; CI, confidence interval; RR, relative risk; 2PN, two-pronuclear; * *P*-value <0.05.
